# Structural Elucidation of Nucleophilic Compounds through Synergistic Coordination and Hydrogen Bonding in a Metal–Organic Framework

**DOI:** 10.1021/jacs.5c07192

**Published:** 2025-07-31

**Authors:** Tomoki Nakagawa, Yuki Wada, Bun Chan, Taichi Baba, Kengo Hanaya, Yuta Koseki, Ryuji Asano, Katsuyuki Aoki, Pavel M. Usov, Masaki Kawano

**Affiliations:** † Department of Chemistry, School of Science, 13290 Institute of Science Tokyo, 2-12-1 Ookayama, Meguro-ku, Tokyo 152-8550, Japan; ‡ TEKMOF Co., Ltd., INDEST, 3-3-6 Shibaura, Minato-ku, Tokyo 108-0023, Japan; § Graduate School of Engineering, 12961 Nagasaki University, Bunkyo 1-14, Nagasaki-shi, Nagasaki 852-8521, Japan; ∥ Faculty of Pharmacy, 12869 Keio University, 1-5-30 Shibakoen, Minato-ku, Tokyo 105-8512, Japan; ⊥ Tsumura Botanical Raw Materials Research Laboratories, Tsumura & Co., 3586, Yoshiwara, Ami-machi, Inashiki-gun, Ibaraki 300-1192, Japan

## Abstract

This study presents the development of a novel metal–organic framework (MOF) denoted as APF-80, suitable for the structural analysis of nucleophilic compounds that have traditionally been challenging to analyze using the crystalline sponge method. It was synthesized using a new mixed-substituent hexaazaphenalene ligand (344-TPHAP) featuring both 3-pyridyl and 4-pyridyl groups. This framework demonstrated remarkable stability toward nucleophilic molecules, which could be captured inside the pores through a synergistic combination of coordination and hydrogen-bonding interactions. APF-80 was successfully applied to determine the structures of 12 bioactive molecules, including naturally occurring alkaloids and pharmaceutical compounds. The host–guest interaction modes observed inside the resulting structures were classified into five distinct types. Binding energy calculations revealed that the guest site energies positively correlated with the crystallographic occupancies and the corresponding interaction types. This multimodal capture mechanism enabled the precise structural analysis of nucleophilic compounds under mild conditions, expanding the scope of molecules that can be analyzed by the crystalline sponge method.

## Graphical abstract

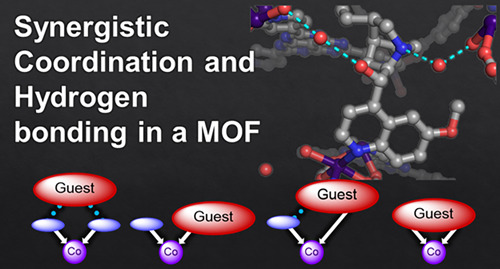

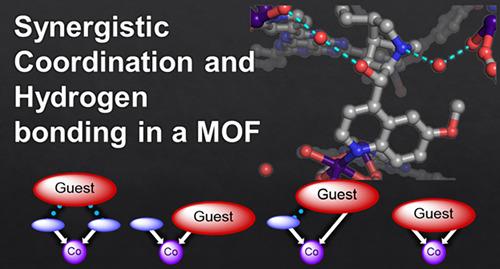

## Introduction

Alkaloids are a diverse group of nitrogen-containing organic compounds widely distributed in nature. Due to their structural diversity and potent biological activities, they play pivotal roles in pharmaceutical development and life science research.^1–4^ While these compounds are primarily derived from plants, they are also found in certain animals and microorganisms,^5^ where they are believed to serve crucial functions in biological defense mechanisms and interspecies communication.^6,7^ Alkaloids typically possess polycyclic structures containing at least one nitrogen atom, which usually exhibits basic and nucleophilic properties serving as the origin of the term “alkaloid”.^8^ These molecules can demonstrate extremely high biological activities, capable of inducing significant physiological effects, even in minute quantities. Well-known examples include morphine, quinine, nicotine, and caffeine. Many alkaloids exist in extremely low concentrations in nature, which complicates the isolation of these compounds in sufficient quantities for further characterization. Moreover, they often feature complex three-dimensional structures with multiple chiral centers, making the accurate determination of their stereochemical structures crucial for drug discovery research. Because of these factors, complete structural determination using conventional analytical methods, such as nuclear magnetic resonance (NMR) spectroscopy and mass spectrometry (MS), is highly challenging. For small- and medium-sized organic molecules, the widely available diffraction-based methods are capable of accurately determining the arrangement of atoms. However, sufficiently large crystals are required to produce the necessary diffraction spots. In the case of single-crystal X-ray diffraction (SCXRD), the minimum size is on the order of 1–10 μm depending on the constituent elements, crystal density, crystallinity, and the diffraction instrument. Growing such single crystals is a nontrivial task with no upper bound on the time and effort it might take to screen and optimize the crystallization conditions. Powder X-ray diffraction (PXRD) and microcrystal electron diffraction (micro-ED)^9–14^ have less stringent requirements for the crystal size and quality. However, the target molecules still need to be crystallographically ordered to provide structural information, which excludes a wide range of compounds.

In the past decade, several methods have been developed that can significantly expedite the crystallization process or even avoid it entirely.^15^ One of the approaches involves crystal growth in the presence of cocrystallization agents, which are discrete species that display a high propensity to form highly ordered inclusion complexes with target molecules.^16–23^ Alternatively, a preformed porous framework can be used as a crystalline matrix, which can align and visualize the encapsulated guest molecules. Several host materials have been tested for this purpose, including metal–organic frameworks (MOFs),^24–41^ hydrogen-bonded organic frameworks (HOFs),^42–46^ and other porous compounds.^47–54^ This technique was originally reported by Fujita and co-workers in 2013 ^26^ who utilized [(ZnI_2_)_3_(TPT)_2_] (ZnI_2_-TPT, TPT = 2,4,6-tri­(4-pyridyl)-1,3,5-triazine) as a crystalline support matrix and named it the crystalline sponge (CS) method. This breakthrough permitted the application of diffraction techniques to the structural analysis of compounds that were traditionally challenging to crystallize, including amorphous solids,^39^ oil compounds,^41,55–57^ and trace quantity samples.^41,58–62^

Despite the growing number of structures solved using the CS method, highly nucleophilic compounds, such as alkaloids, have been largely incompatible with the host frameworks because guests tend to damage MOF crystals.^63^ Various solutions to this problem have been proposed, including carrying out guest encapsulation for a longer time at lower temperatures^64^ and formation of ion pairs that shield the nucleophilic sites.^65^ However, these approaches have narrow applicability and suffer from drawbacks of their own. For example, lowering the encapsulation temperature could lead to a marked decrease in the success rates of crystallographic resolution.^66^ Therefore, to find a more broadly applicable solution, it is imperative to continue developing new crystalline sponges with improved resistance to nucleophilic compounds.

In some instances, the nucleophilic nature could be beneficial for the molecular alignment inside the framework pores through coordination with open metal sites.^29,38,67^ While showing some success, this strategy is inherently limited to substituents that can generate sufficiently robust coordination bonds such as carboxylate or pyrazolate. Moreover, due to the overreliance on a strong single-point binding without additional supporting interactions, the vibrational and rotational motion of the molecule might not be adequately restricted. As a result, some parts could experience severe disorder, thus lowering the diffraction data quality and reducing the reliability of the structure model.

In the design of MOFs, the orientation of coordinating groups around the ligand core strongly influences their structural properties, such as symmetry, topology, and flexibility, as well as the size and shape of the pores. In our previous studies, we have extensively utilized ligands based on 1,3,4,6,7,9-hexaazaphenalene (HAP) core decorated with either 4-pyridyl (4-TPHAP, **4-L**)^68–70^ or 3-pyridyl (3-TPHAP, **3-L**)^41,71^ groups for the construction and characterization of a wide variety of frameworks. These studies provided useful insights into the relationship between the pyridyl group orientation and the assembled structures. **4-L** possesses high symmetry and regular coordination directions, which are not dependent on the rotation of 4-pyridyl groups, allowing it to serve as a triangular connecting unit.^72,73^ Due to the stable predictable geometry, a large number of MOFs could be generated by combining this ligand with different metal ions.^68^ In contrast, **3-L** can assume a range of different coordination arrangements depending on the relative rotation of the 3-pyridyl groups. Because of this conformational freedom, the incorporation of **3-L** into ordered framework structures was found to be difficult to achieve. Only one MOF based on this ligand has been reported to date, Co-3-TPHAP (also referred to as APF-1, adaptable porous framework), which was comprised of Co^2+^ ions and also contained 1,4-benzenedicarboxylate (BDC) as a coligand helping to stabilize the structure. In this framework, the bent coordination angle of 3-pyridyl groups brought the Co^2+^ cluster closer to the HAP core facilitating the formation of a chelated hydrogen-bonded water site (Figure 1b),^41^ which was found to be highly efficient at capturing and aligning guest molecules. APF-1 was then applied as a crystalline sponge to determine the structures of 14 different bioactive molecules down to atomic resolution (electron density could be unambiguously correlated with the molecular structure for at least one crystallographic site) and the minimal use of restraints and constraints during the refinement.

**Figure 1:**
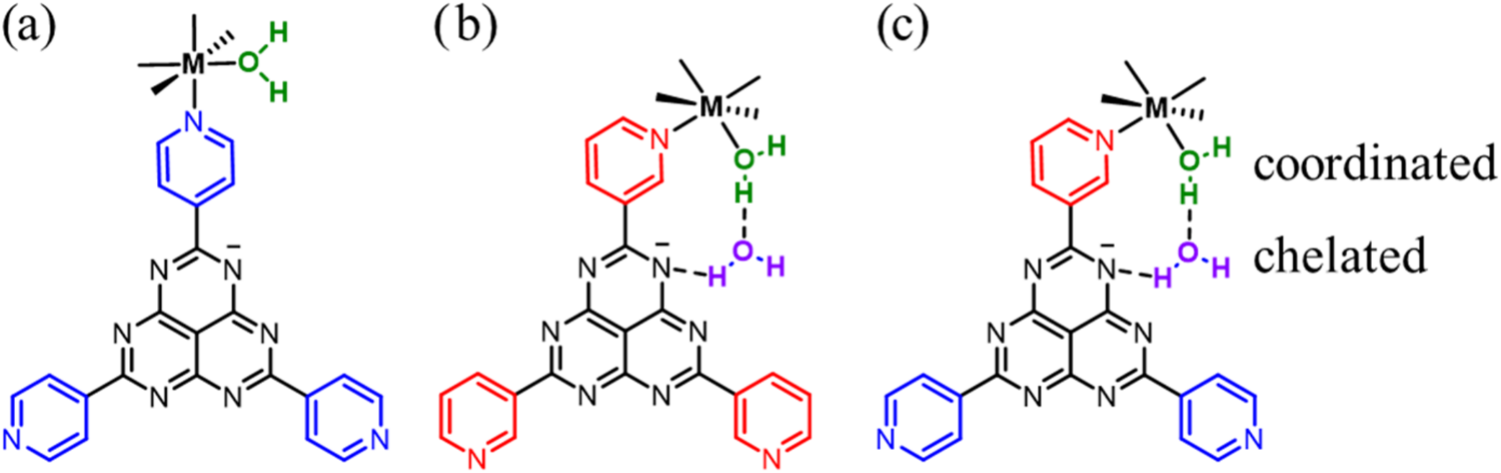
Chemical structures of HAP-based ligands showing their coordination modes inside MOFs and the resulting interactions with water molecules: (a) **4-L**, the distance between the coordinated water and the HAP skeleton is too long for cooperative interactions; (b) **3-L**, a hydrogen-bonded chelation site for water molecules is formed due to closer proximity of the metal center and the HAP core; and (c) **344-L**, the same chelation site is also formed.

To combine the interactivity of **3-L** and the well-defined symmetry of **4-L**, a new ligand featuring mixed pyridyl substituents was developed, 2-(3-pyridyl)-5,8-di­(4-pyridyl)-1,3,4,6,7,9-hexaazaphenalene (344-TPHAP, **344-L**). It was more conformationally restricted compared to the 3-pyridyl analogue but was still able to create the chelated hydrogen-bonded water site that is important for interacting with guest molecules (Figure 1c). **344-L** was reacted with CoBr_2_ and BDC coligand under solvothermal conditions to provide a new MOF denoted as APF-80. One of the Co^2+^ centers featured two open metal sites next to each other, which were coordinated by labile *N,N*′-dimethylacetamide (DMA) synthesis solvent molecules in the as-synthesized crystal. As a result, it was theorized that this dual open site could capture nucleophilic compounds by coordination to Co^2+^, similar to the previously reported coordinative alignment strategy.^29^ These kinds of molecules are often difficult to analyze by the crystalline sponge method since they can damage the framework crystals.^64^ 12 compounds featuring nucleophilic groups, including nitrogen-based heterocycles, amine, alcohol, sulfoxide, and sulfonamide, were encapsulated inside this framework to test its performance as a crystalline sponge. Structural elucidation revealed that these guests were immobilized by a combination of coordination to the Co^2+^ centers as well as hydrogen bonding with the HAP core and interstitial water molecules. In particular, the hydrogen-bonded water network formed a series of interactions with the guest molecules, providing well-defined crystallographic sites in a similar fashion to APF-1. Based on these results, the ability of APF-80 to withstand the damaging nucleophilic substituents in a variety of encapsulated molecules while still allowing it to visualize their structures was confirmed, thus expanding the range of functional groups analyzable by the crystalline sponge technique.

## Results and Discussion

### Synthesis and Characterization

The synthesis of HAP is typically performed using a one-pot condensation reaction between tricyanomethanide anion and substituted amidino precursors under relatively forcing conditions (high temperature, excess amidino).^68,71,74^ While this reaction could provide a variety of HAP derivatives with the same substituent in good yields using solid-state synthesis (Scheme 1), it is unsuitable for the attachment of multiple different functional groups around the same core. Previous work on the HAP condensation reaction pathway identified several intermediary species, including 4,6-diamino-5-cyanopyrimidine and diaminopyrimidinopyrimidine.^75–77^ Based on these results, a two-step synthetic route was considered where the pyrimidine intermediate substituted with a 3-pyridyl group was first obtained by reacting tricyanomethanide with one equivalent of the corresponding amidino precursor (Figures S1–S3 and S8). Then, this compound was combined with 4-amidinopyridine to complete the HAP core. Using this synthetic strategy (Scheme 1), we successfully prepared a new mixed-substituent tripyridyl HAP ligand, 2-(3-pyridyl)-5,8-di­(4-pyridyl)-1,3,4,6,7,9-hexaazaphenalene (denoted as 344-TPHAP, **344-L**). The final compound was isolated as a tetraphenylphosphonium (TPP) salt in a moderate yield, and its structure was confirmed by spectroscopic analysis (Figures S4–S7, S9, and S10).

**Scheme 1:**
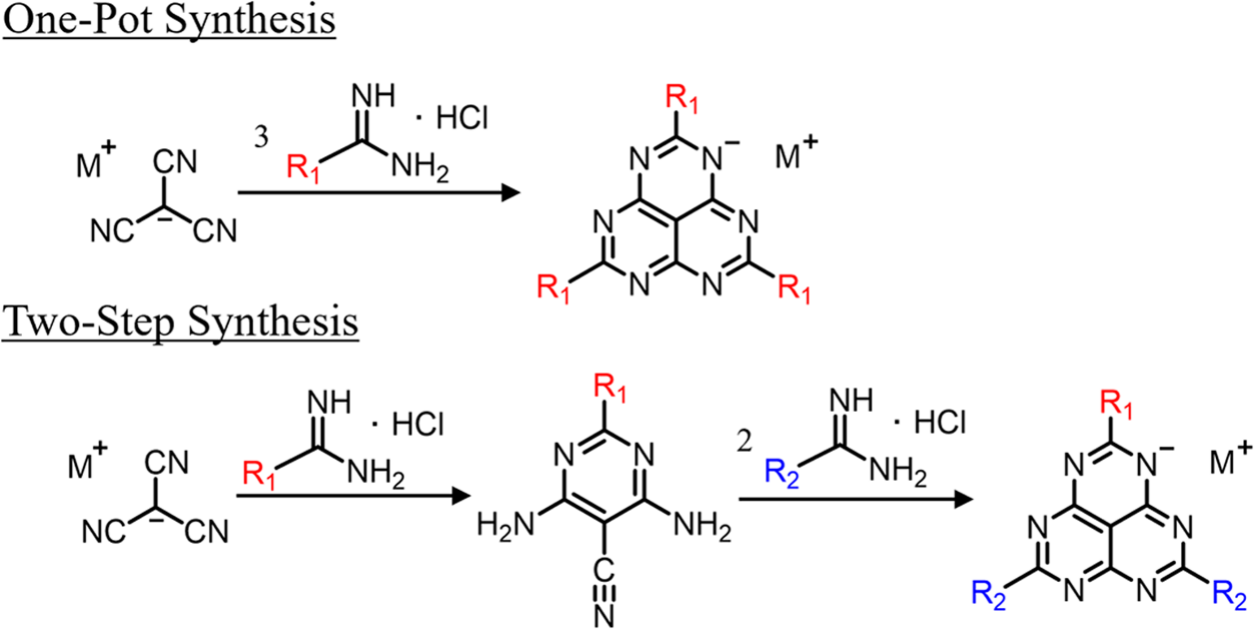
General Syntheses of Substituted HAP Analogues

Building on the success of employing **3-L** to construct APF-1, the framework assembly was also carried out with **344-L** using a similar synthetic approach. The cooperative interactions between the HAP core and the two labile solvent sites on the Co^2+^ nodes have proven to be highly efficient at promoting the ordering of internal hydrogen-bonded water networks. Reacting **344-L** with CoBr_2_ and BDC in DMA under solvothermal conditions resulted in the appearance of magenta prismatic crystals. They were analyzed by single-crystal X-ray diffraction (SCXRD), which showed a three-dimensional framework crystallized in an orthorhombic *Pbca* space group with the molecular formula [Co_4_(**344-L**)_2_(BDC)_3_(DMA)_4_] (denoted as APF-80). The structure consisted of asymmetric Co^2+^ dimers with both metal centers existing in distorted octahedral coordination environments, which were connected by three carboxylates, as well as one 3-pyridyl and two 4-pyridyl groups from **344-L** (Figure 2a). The connectivity of carboxylate groups was identical to that of the Co^2+^ dimer in APF-1, giving rise to a 3-connected Co-carboxylate node. As a result, the underlying Co-carboxylate network displayed a (6,3) connected net topology. On the other hand, the relative arrangement of pyridyl groups was altered, which caused both DMA molecules to coordinate to only one Co^2+^ center (Co2 in Figure 2b) rather than one solvent molecule on each Co^2+^ center observed in APF-1. APF-80 exhibited an open pore space with the smallest aperture openings of 4.36 Å and a maximum diameter of 8.33 Å, as calculated using the pore analyzer function in the Mercury program. These pores were interconnected along the *a*-axis and *c*-axis directions.

**Figure 2:**
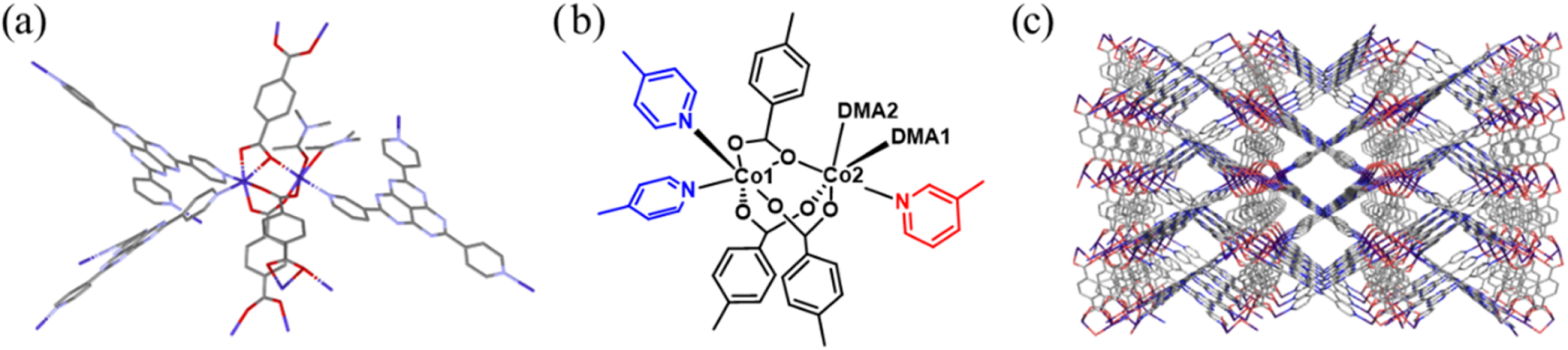
Crystal structure of APF-80: (a) structure of the Co^2+^ dimer and the surrounding ligands; (b) chemical representation of the Co^2+^ dimer, blue and red colors show 4-pyridyl and 3-pyridyl groups, respectively; and (c) view of the three-dimensional structure along the *a*-axis showing open pores. Co, purple; C, gray; O, red; and N, blue. Hydrogen atoms were omitted for clarity.

The properties of isolated APF-80 were additionally characterized using various spectroscopic techniques and physical measurements. The ultraviolet–visible (UV–vis) diffuse reflectance spectrum of the MOF closely resembled that of the free **344-L**, especially in the high-energy region (<400 nm), indicating similar energy transitions (Figure S7). There were also additional lower intensity bands observed between 400 and 600 nm, which were attributed to the d–d transition of the Co^2+^ centers. The infrared (IR) spectrum of APF-80 exhibited several characteristic vibrational bands due to the presence of organic ligands and solvent in its structure (Figures S9 and S10). The phase purity of the synthesized MOF crystals was confirmed by performing powder X-ray diffraction (PXRD) measurement inside a glass capillary filled with DMA solvent. The obtained data (Figure S11) showed a good match with the pattern predicted from the single-crystal structure, which was additionally supported by the Le Bail refinement (Figure S12). Thermogravimetric analysis coupled with differential scanning calorimetry (TGA-DSC) was measured to investigate the thermal stability of APF-80 (Figure S13). The weight loss steps below 200 °C were attributed to the evaporation of interstitial solvent molecules (water and DMA). After that, a stable plateau was observed until 400 °C. Above that temperature, ligand degradation and final framework breakdown had occurred.

### Guest Encapsulation and Structural Analysis

To assess the ability of APF-80 to crystallographically visualize guest molecules, artemisinin was used as a test molecule, as it could be compared with previous encapsulation studies inside APF-1. The multiple oxygen atoms present in the structure of artemisinin can function as interactive sites, allowing it to probe the pore environment (Figure 3). Its encapsulation into APF-80 caused the framework space group to transform from the centrosymmetric *Pbca* to the noncentrosymmetric *P*2_1_2_1_2_1_, with a Flack parameter of 3.4(3)%, which was sufficiently reliable for absolute structure determination. Five distinct artemisinin crystallographic sites were identified within the asymmetric unit, four of which involved hydrogen bonding with coordinated and chelated water molecules. The last site displayed coordination of the lactone group to the open Co^2+^ center (Figure 3). Interestingly, the occupancy of the coordinated artemisinin molecule was only 16.4% compared to 28.0–46.1% observed for hydrogen-bonded sites, suggesting that its overall interactions with the host framework were less favorable (*vide infra*). The guest occupancies in APF-80 were notably lower than in the previously reported structure of APF-1 containing artemisinin (several sites at 100%) encapsulated under the same conditions.^41^ This discrepancy can be explained by the smaller diameter of the narrowest pore aperture in APF-80 (4.36 Å) compared to that of APF-1 (7.16 Å), which limited the diffusion of guest molecules throughout the crystal. Nevertheless, despite the lower occupancy, the structure of artemisinin could still be clearly visualized down to the atomic resolution for site A (Figure S14). This result confirmed the ability of APF-80 to serve as a crystalline sponge, thus prompting us to extend the encapsulation studies to include a wider range of bioactive compounds and further evaluate the framework performance.

**Figure 3:**
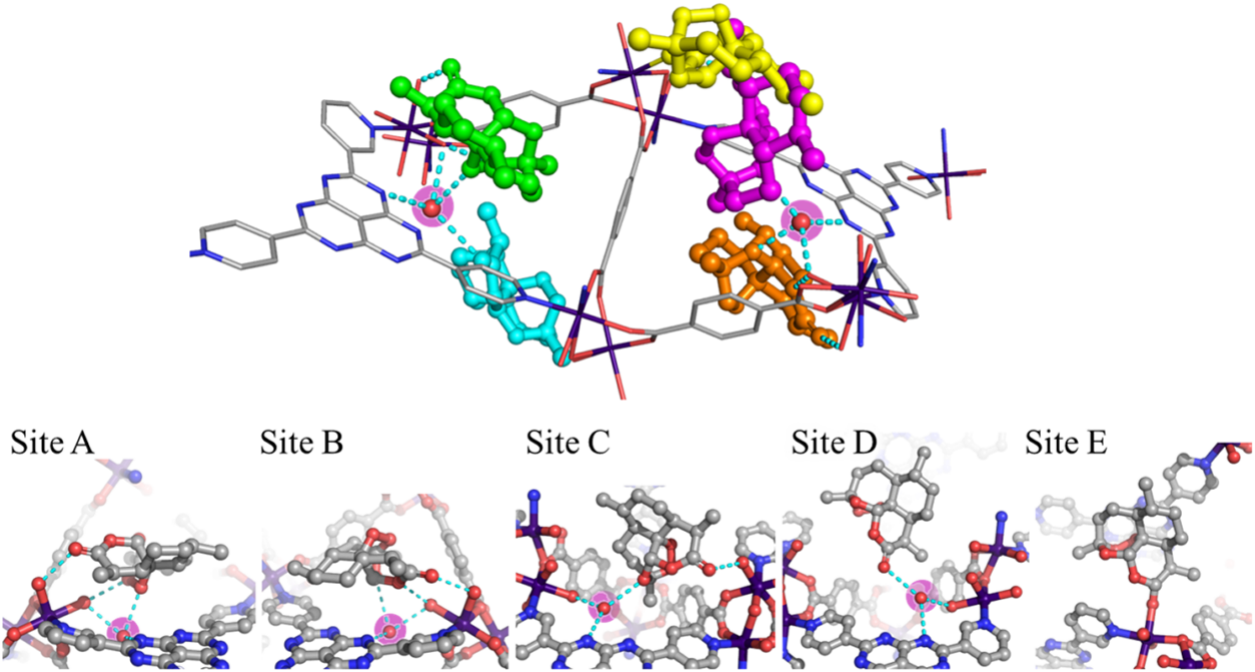
Hydrogen-bonding interactions between encapsulated artemisinin and internal water molecules inside APF-80. Overview of all of the sites (*upper*) (site A, light green; site B, orange; site C, magenta; site D, cyan; site E, yellow). Hydrogen-bonding interactions of each individual site (*lower*). The water sites chelated between the HAP core and a water molecule coordinated to Co^2+^ were highlighted in purple. Cyan dash lines represent hydrogen bond contacts. Co, purple; C, gray; O, red; and N, blue. Hydrogen atoms were omitted for clarity.

Encapsulation studies were performed on a diverse range of bioactive compounds containing nucleophilic substituents, with a focus on alkaloids, using either pure *n*-heptane or 10% acetone/*n*-heptane mixture (Table S1). X-ray diffraction analysis of the resulting crystals revealed an additional 11 structures inside APF-80 (Figure 4). One of the molecules was a synthetic intermediate whose relative stereochemistry was previously unknown. For most structures, the application of crystallographic restraints or constraints was not required to obtain reasonable models for at least one crystallographic site (Table S2 and Figure S28). Furthermore, the observed electron densities could be unambiguously matched with each atom in the guest molecule, indicating an atomic resolution level analysis (Figure 4).

**Figure 4:**
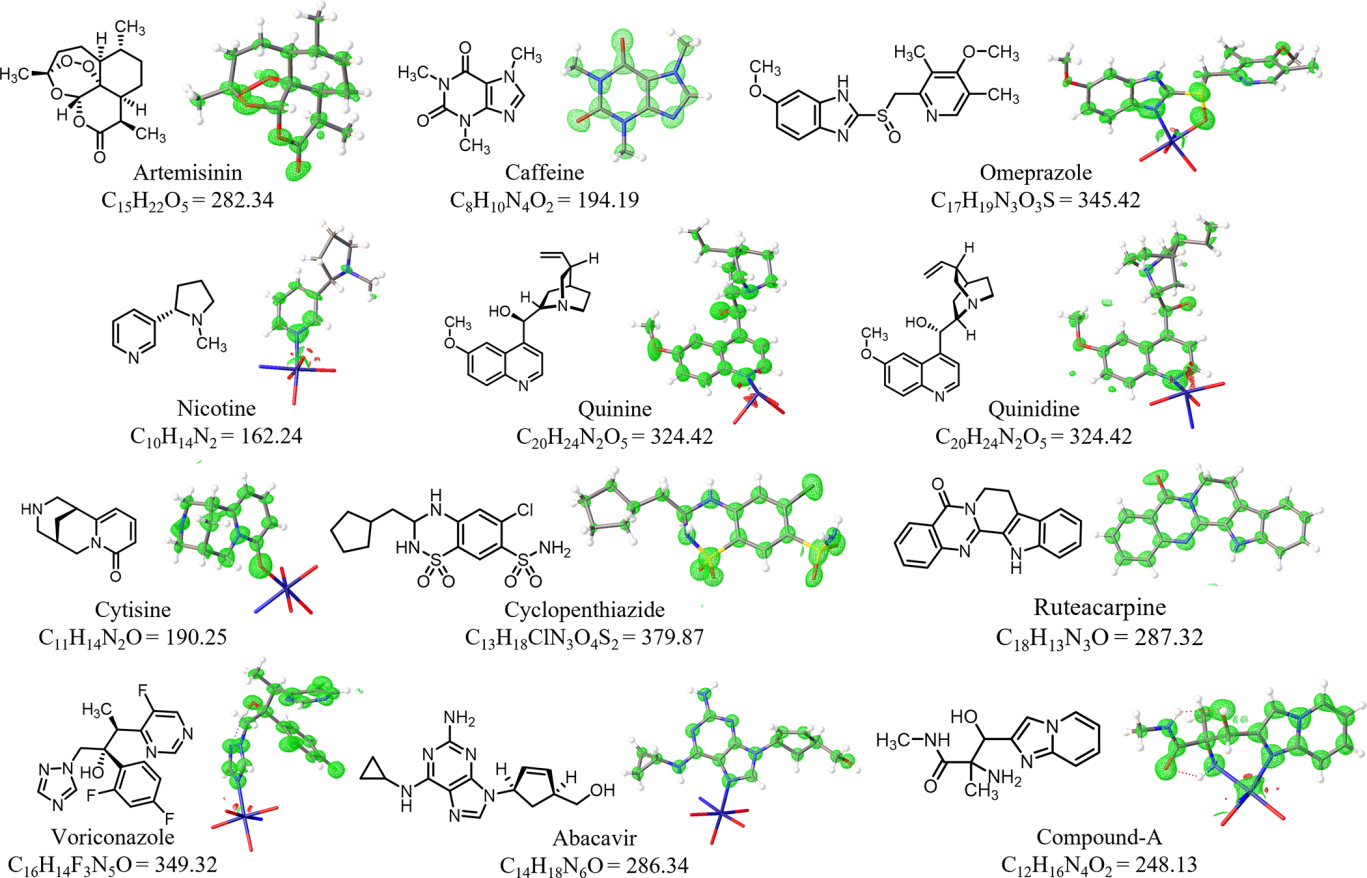
Electron density maps (2Fo–Fc) of elucidated structures of nucleophilic guests encapsulated in APF-80. The corresponding molecular formulas and molar masses (g mol^–1^) are shown. Omeprazole, cyclopenthiazide, and compound-A were encapsulated as racemic mixtures.

Previous studies regarding the structural analysis of nucleophilic compounds using ZnX_2_-TPT (X = Cl or I) crystalline sponge demonstrated that the host crystal had to be soaked in a guest solution for a long time (7 days) at low temperatures (4 °C) to avoid the framework damage.^64^ This strategy was only partially successful since nicotine ended up destroying the crystal even under these mild encapsulation conditions, thus precluding its structural analysis. In another report, the encapsulation temperature was found to strongly influence the guest uptake process with higher temperatures positively correlating with the success rate of visualizing the guest inside the pore.^66^ Because of these opposing temperature trends, the structural analysis of highly nucleophilic compounds using the crystalline sponge method presents a great challenge.

Because of the presence of open metal sites in APF-80, it was theorized that they could act as interactive sites for nucleophilic groups, thus facilitating their ordering inside the pore. To test this, three nucleophilic compounds, namely, caffeine, omeprazole and nicotine, were encapsulated, and the resulting host–guest crystals were analyzed by SCXRD (Figure 5). No degradation of APF-80 crystals was observed in the presence of these compounds even at 40 °C, suggesting that the framework exhibited an improved stability. The structure of caffeine revealed that it was incorporated at seven crystallographic sites (Figure S15), most of which (sites A–D) were supported primarily through hydrogen bonding with coordinated water molecules. Furthermore, pairs of caffeine molecules formed π–π stacks together with the BDC ligands (Figure S16). A combination of these interactions permitted the pore environment to effectively restrict the rotational disorder (Figure S17). The space group of the omeprazole-loaded APF-80 changed to *Pca*2_1_, suggesting that both enantiomers were captured by the framework. While selective encapsulation of only one enantiomer was observed in the analyzed structure, the presence of glide planes implied that the opposite enantiomer was also present in the opposite chirality pores resulting in an overall racemic crystal. This result correlated with previous observations of encapsulation of racemic mixtures into crystalline sponges.^41,58^ The guest molecules were located at three crystallographic sites (Figure S18) and were chelated via the benzimidazole nitrogen and the sulfoxide oxygen atoms to a Co^2+^ center featuring two labile positions. This result demonstrated the ability of APF-80 pore to accommodate chelating compounds without compromising the structural integrity of the framework. Due to its pyridine ring and relatively small size, nicotine is a highly damaging guest, but it could still be encapsulated inside APF-80 and resolved crystallographically. Nicotine molecules were coordinated to Co^2+^ centers, which sufficiently restrained the molecular motion of the pyridine ring to observe the electron density with atomic resolution (Figure S19). However, the *N*-methyl pyrrolidine part lacked any interactions with the framework and therefore was more conformationally flexible, leading to a diffuse electron density. In addition, one of the sites (site C) was only supported by weak hydrophobic interactions. The main reason for the incompatibility of nucleophilic compounds with crystalline sponge hosts is the ability of their functional groups to coordinate to metal centers and displace the structure-supporting ligand, thus causing their structural collapse. The improved stability of APF-80 can be rationalized by the presence of anionic charge on both of its ligands, **344-L** and BDC. Any ligand exchange reactions between the framework and neutral nucleophilic molecules would be unfavorable because they would release charged species into a low-polarity environment (*n*-heptane) present during the encapsulation. Moreover, the two neighboring labile sites in the Co dimer could efficiently capture strongly coordinating groups, which mitigated the negative impact of those groups on the structural integrity. Because of these beneficial features, APF-80 has the potential to serve as an effective crystalline sponge host for the structural analysis of nucleophilic compounds.

**Figure 5:**
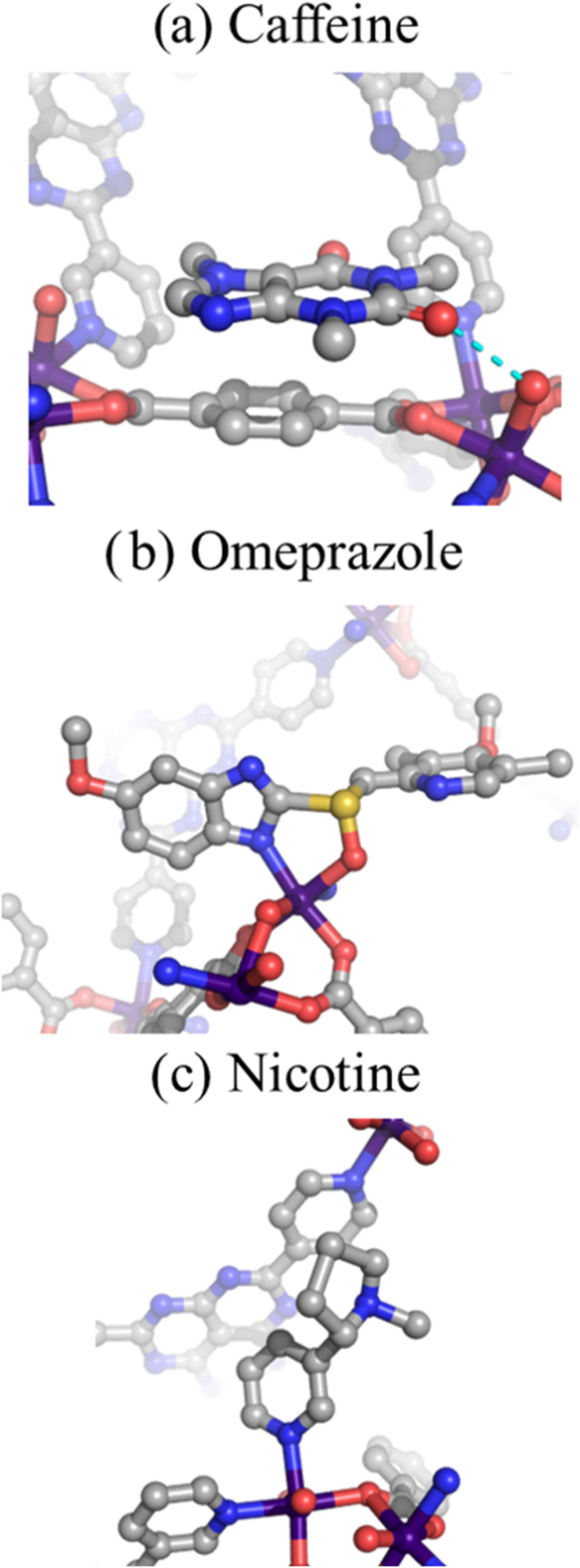
Structures and interactions of encapsulated guests inside APF-80: (a) caffeine, (b) omeprazole, and (c) nicotine. Cyan dash lines represent hydrogen bond contacts. Co, purple; C, gray; O, red; N, blue; and S, yellow. Hydrogen atoms were omitted for clarity.

For a framework to be usable as a crystalline sponge, it must be able to reveal small differences between chemically and structurally similar molecules with minimal ambiguity. To assess the ability of APF-80 for this task, the encapsulation of quinine and quinidine, which are a diastereomeric pair, was investigated. These compounds differ in the stereochemical configuration of the tertiary amine group and hydroxyl group adjacent to the quinoline ring. However, this subtle structural difference translates into a pronounced change to their pharmacological effects. Specifically, quinine functions as an antimalarial drug,^78^ while quinidine acts as an antiarrhythmic medication.^79^ Both compounds were successfully encapsulated into APF-80, leading to the change of the space group to the noncentrosymmetric *P*2_1_2_1_2_1_. Electron density maps clearly showed the arrangement of hydroxyl groups, confirming their absolute chirality with Flack parameters of 4.4(2)% and 12.8(3)% for quinine and quinidine, respectively. In the structure of quinine-loaded framework, the guest occupied two distinct sites within the asymmetric unit (Figure S20). One of the sites displayed coordination of the quinoline heterocycle to the Co^2+^ center and hydrogen bonding between the HAP core and the hydroxyl group. Whereas the second quinine molecule was supported entirely by hydrogen bonding. In comparison, quinidine was encapsulated at only one crystallographic site, which also featured a coordinated quinoline moiety (Figure S21). The additional hydrogen-bonding interactions formed a complex network involving several coordinated and chelated water molecules (Figure 6). It was interesting to compare the quinine and quinidine sites that were supported by coordination bonds. Detailed structural analysis revealed significant differences in the angles between the Co–N coordination bond and the plane of the quinoline heterocycle (Figure 6). Specifically, quinine exhibited a lower deviation from a linear coordination with an angle of 27.5(2)°, compared to a more bent angle of 43.3(3)° for quinidine. The length of corresponding coordination bonds was also altered, 2.204(5) and 2.351(5) Å for quinine and quinidine, respectively, which indicated that an increase in angular distortion was accompanied by bond weakening. The typical Co–N coordination bonds in quinoline-containing complexes have lengths of around 2.077 Å, as obtained from the structures in the CCDC crystallographic database (Table S3). The cause of the angle distortion can be understood by considering the other interactions that occur on the opposite side of the guest molecules. In particular, because of the hydrogen-bonded network that formed between the hydroxyl and tertiary amine groups of quinidine, the interstitial water molecules and the HAP core (Figure 6), the entire guest molecule was pulled into the pore, thus diminishing its interaction with the Co^2+^ centers. The hydrogen bonding was less pronounced in the structure of encapsulated quinine allowing it to form a stronger more linear coordination bond. This result revealed that even though the quinidine molecule was placed in a destabilized position due to the competing coordination and hydrogen-bonding interactions, it was still sufficiently immobilized to be resolved by X-ray crystallography down to atomic resolution with minimal application of restraints and constraints. The encapsulation behavior of APF-80 was akin to protein binding by small molecules where slight alteration to a molecular structure of the binding partner could cause dramatic modifications to the downstream physiological effects. It was facilitated by a delicate balance between guest interactions with the hydrogen-bonded water network and the formation of stronger coordination bonds. In this sense, the pore of APF-80 acted as an adaptable enzyme binding pocket, adjusting its interaction modes to accommodate different molecular features and permitting it to differentiate even closely related compounds.

**Figure 6:**
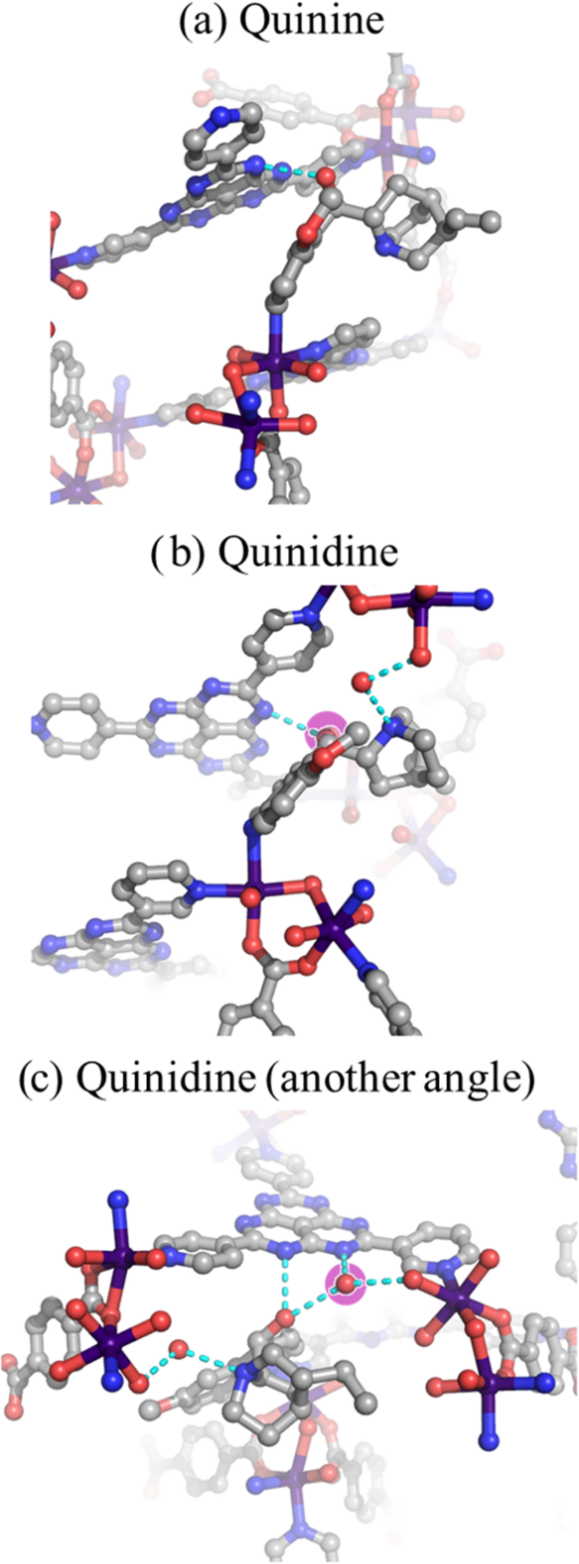
Structures and interactions of encapsulated guests inside APF-80: (a) quinine and (b) quinidine highlighting the coordination of the quinoline moiety to Co^2+^, and (c) a different viewing angle of quinidine focusing on hydrogen bonding. The water sites chelated between the HAP core and a water molecule coordinated to Co^2+^ were highlighted in purple. Cyan dashed lines represent hydrogen bond contacts. Co, purple; C, gray; O, red; and N, blue. Hydrogen atoms were omitted for clarity.

To further test the tolerance of APF-80 toward nucleophilic substituents, the encapsulation study was expanded to include a wider range of pharmaceutically active compounds. An additional five molecules were encapsulated and analyzed by X-ray diffraction, namely, cytisine, cyclopenthiazide, rutaecarpine, voriconazole, and abacavir (Figure 7). Their structures feature a variety of nucleophilic groups that could detrimentally affect the host framework through ligand displacement, including amino, hydroxyl, triazole, pyrimidine, and sulfonamide. All five compounds were included in APF-80 and crystallographically visualized inside the resulting host–guest structures, further confirming the structural stability of the framework. Cytisine was incorporated at four crystallographic sites (Figure S22) supported by either direct coordination to Co^2+^ centers or hydrogen bonding with the surrounding water molecules. Both the carbonyl oxygen of the fused pyridone (sites A and B) and the secondary amine (site C) were able to form a coordination bond. The occupancies of sites C and E were fixed at 40 and 30%, respectively, due to the overlapping electron density from the disordered solvent molecules. In the cyclopenthiazide-loaded crystal, the space group remained as the achiral *Pbca* with both enantiomers related by a glide plane visible inside the unit cell. Encapsulated structures of cyclopenthiazide and rutaecarpine did not form any coordination bonds. In the former case, the guest molecules were captured by hydrogen bonding of sulfonamide and amino groups with the HAP core and water molecules (Figure S23). For rutaecarpine, the lack of coordination bonds was especially unusual, since it contained a chelating pocket between pyrrole and pyrimidone groups, which was expected to be highly suitable for binding with the dual labile site around one of the Co^2+^ ions. Instead, the guest sites were immobilized through hydrogen bonding with water molecules (Figure S24). This behavior was attributed to the bulky nature of the encapsulated compounds, which prevented the coordinating groups from favorably accessing the Co^2+^ centers. The last two compounds of this series, voriconazole and abacavir, were composed of nitrogen-based heterocycles featuring several nucleophilic sites. After encapsulation into APF-80, the triazole and purine rings in their respective molecules were coordinated to the Co^2+^ centers (Figures S25 and S26). In the case of abacavir, an additional hydrogen bond was formed between the secondary amino group and a coordinated acetone molecule, giving it the appearance of a chelated binding. Looking closer at the interactions between the guests and the framework, another unexpected tendency was uncovered. The Co dimer possesses a nonsuperimposable mirror image; however, the parent APF-80 crystallized in the centrosymmetric space group *Pbca* in which both enantiomorphs were present in equal numbers, resulting in an overall achiral structure. This structural feature was similar to that of previously reported APF-1, where a chiral recognition phenomenon was observed with certain guests. In the present study, voriconazole and abacavir were encapsulated in their enantiomerically pure forms; however, they generated two crystallographic sites occupying all of the available Co dimer positions. This behavior implied that there was no strong preference for any particular chirally interacting guest–dimer pair. In the context of crystalline sponge applications, the lack of strong chiral recognition could be advantageous since it increases the likelihood of a successful capture of the target molecule regardless of its absolute configuration. The space group of the encapsulated structures was changed to the noncentrosymmetric *P*2_1_2_1_2_1_ with the Flack parameters of 1.7(2)% and 9.2(2)% for voriconazole and abacavir, respectively, which indicated that the guest chirality could still be reliably determined. Moreover, both sites had relatively high occupancy and could be refined with minimized atomic disorder and without the application of any crystallographic restraints or constraints. Overall, the range of nucleophilic compounds that could be analyzed using APF-80 was successfully expanded, demonstrating the framework’s compatibility with a variety of molecular shapes and functional groups.

**Figure 7:**
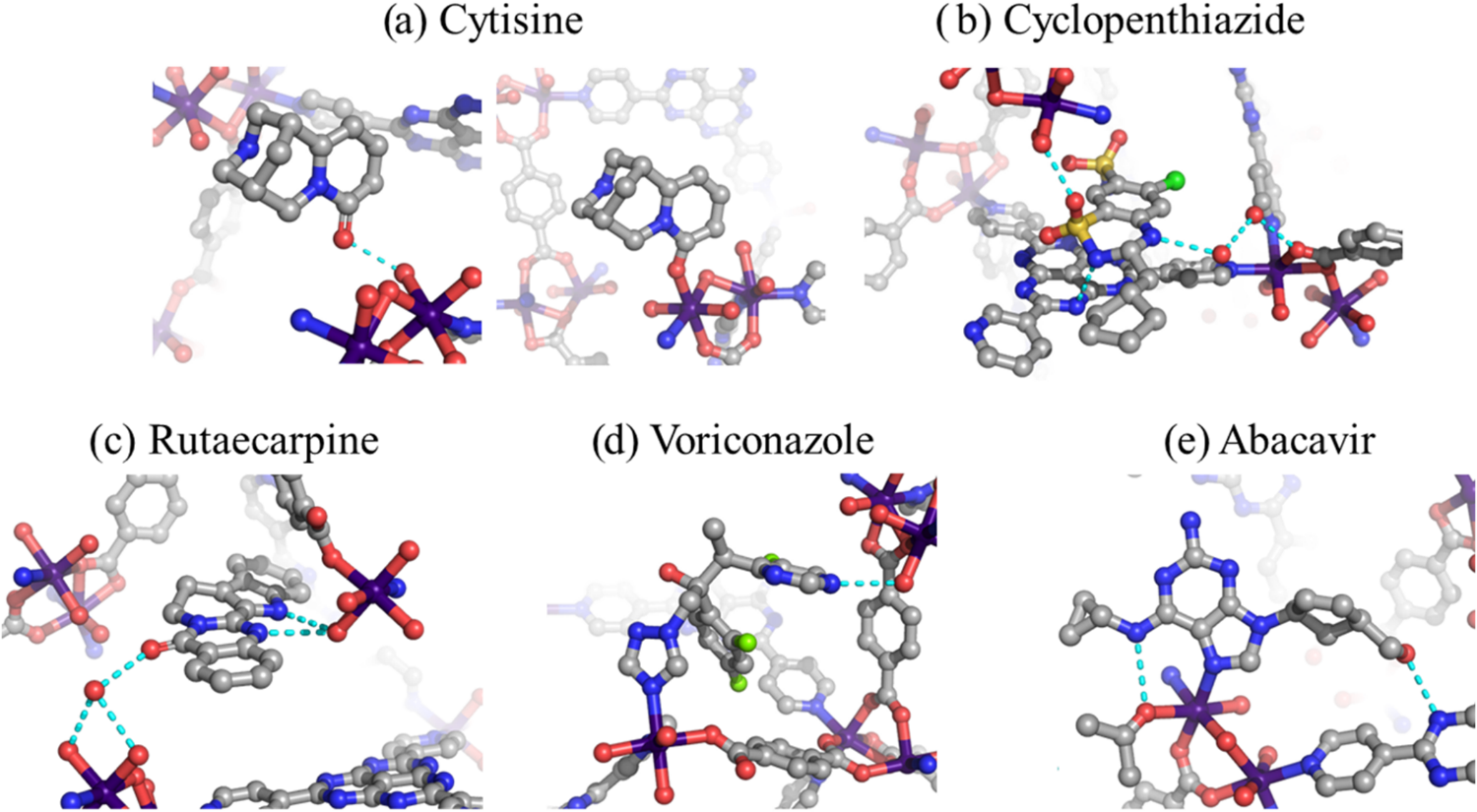
Structures and interactions of encapsulated guests inside APF-80: (a) cytisine, (b) cyclopenthiazide, (c) rutaecarpine, (d) voriconazole, and (e) abacavir. Cyan dash lines represent hydrogen bond contacts. Co, purple; C, gray; O, red; N, blue; S, yellow; and Cl, light green. Hydrogen atoms were omitted for clarity.

Finally, the utility of APF-80 in the structural analysis of previously unreported compounds, especially where the determination of absolute stereochemistry is required, was assessed. A reaction intermediate (referred to as compound-A) composed of imidazo­[1,2-*a*]­pyridine skeleton substituted at the 2 position was used for this study. It contained aromatic and aliphatic nucleophilic nitrogen-based groups. In addition, the molecule possesses two stereocenters, and the encapsulation was performed on a two-component racemic mixture with the goal of evaluating the relative chirality. The space group of APF-80 remained as the achiral *Pbca*, indicating encapsulation of both enantiomers, as observed for omeprazole and cyclopenthiazide. Compound-A was located at three crystallographic sites supported by different interaction modes (Figures 8 and S27). The highest occupancy site (100%, site A) displayed a chelation to the Co^2+^ ion through the imidazole ring and the primary amine, similar to the above-mentioned omeprazole structure. From this structure, the relative arrangement of amino and methyl groups on one stereocenter and hydroxyl group on the neighboring stereocenter could be unambiguously established based on coordination interactions. These substituents are particularly difficult to distinguish based solely on their electron densities. Two additional low occupancy sites (47.7%, site B and 21.7%, site C) were identified. Site B exhibited the same stereochemical configuration as site A, whereas site C was the opposite enantiomer. Several hydrogen bonds between the nucleophilic groups of compound-A, the internal water molecules, and the HAP core supported the guests. Interestingly, one of the hydrogen bonds formed between the imidazole ring of sites B and C and the hydroxyl group of site A, which was akin to the formation of an adsorbed multilayer (Figure 8). This example demonstrated the successful differentiation of similar electron density elements in the guest molecule by examining their hydrogen-bonding and coordination interactions. Because of this interaction versatility inside the APF-80 pore, the relative stereochemistry could be straightforwardly assigned even when encapsulating racemic mixtures.

**Figure 8:**
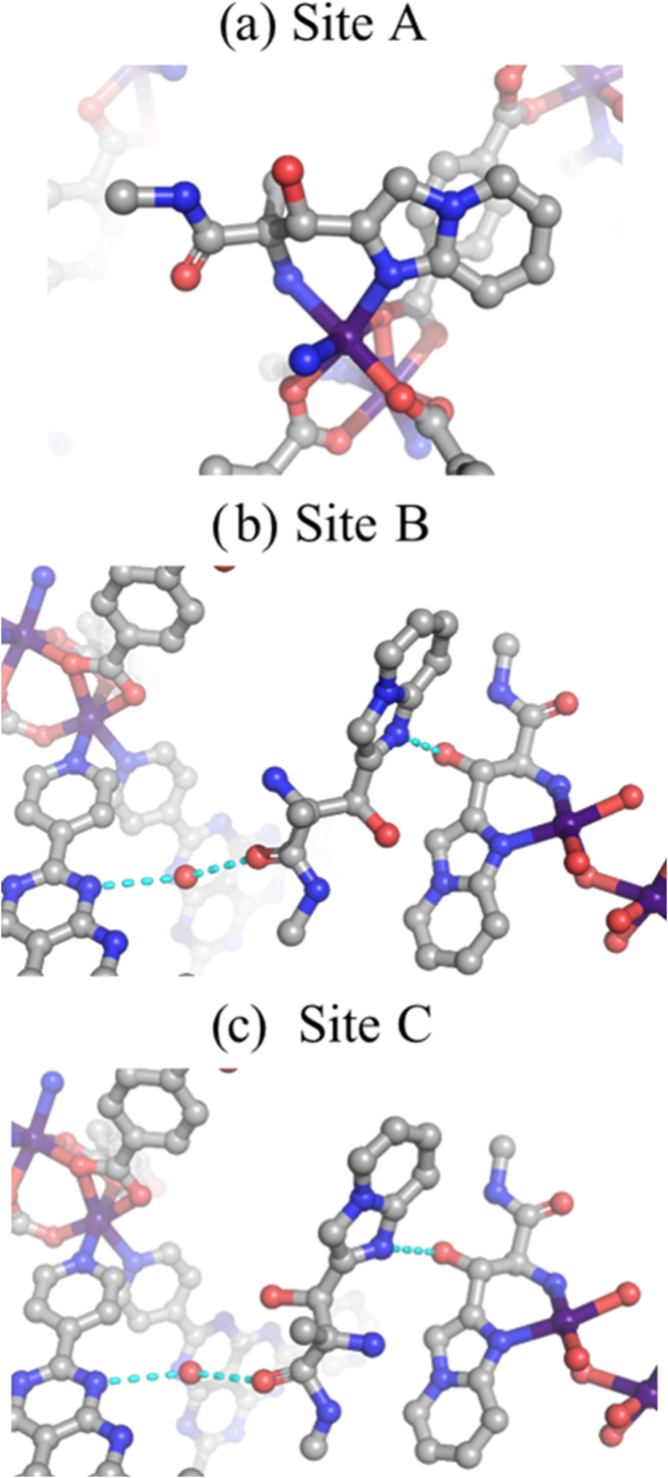
Structure and interactions of encapsulated compound-A inside APF-80: (a) site A, (b) site B, and (c) site C. Cyan dashed lines represent hydrogen bond contacts. Co, purple; C, gray; O, red; and N, blue. Hydrogen atoms were omitted for clarity.

Inspection of the guest structures encapsulated inside APF-80 identified several recurring interaction patterns, which were systematically categorized into five distinct types (Figure 9). Molecules that belong to type I did not display any strong interactions with the framework backbone or internal solvent molecules such as hydrogen or coordination bonds. Instead, they were supported entirely by weaker hydrophobic interactions. Representative examples of type I guests were caffeine (site G) and nicotine (site C). Due to the weakness of these interactions compared to hydrogen or coordination bonds, the guest sites exhibited very low occupancies and a high degree of disorder appearing as diffuse electron densities that were difficult to model. The remaining four types were defined by the guest interactions with the labile sites on the Co^2+^ dimers. Type II interaction mode involved guest immobilization exclusively through hydrogen bonding with coordinated water molecules or the HAP core of the ligand. This type was the most common among the analyzed host–guest structures accounting for 51% of all sites. This prevalence was attributed to the ability of the surrounding water molecules to generate adaptable hydrogen-bonded networks that could effectively capture a wide variety of compounds. Types III–V interaction modes included the formation of coordination bonds between the guest and the Co^2+^ ion. In the case of type III, the guest acted as a monodentate ligand attaching to one of the labile sites around the metal center. At the same time, the neighboring labile site was occupied by a solvent molecule, such as water or acetone, which existed independently and showed no interactions with the guest. Type IV was structurally similar to type III; however, in this case, the solvent and guest molecules coordinated to the same Co^2+^ center formed a hydrogen bond between each other. This interaction mode can be considered a hybrid of hydrogen bonding and coordination and offers greater stabilization to encapsulated compounds (*vide infra*). It required the presence of two nucleophilic sites in close proximity to each other. One example of a guest displaying type IV interactions was abacavir where the purine heterocycle was coordinated to the Co^2+^ ion while the nearby secondary amino group hydrogen-bonded with the coordinated acetone molecule (Figure S26). In the final interaction type observed inside APF-80, type V, the guests acted as bidentate ligands binding to the same Co^2+^ center in a chelated manner. The presence of two labile sites in a *cis* configuration enabled guest capture without compromising the framework’s structural integrity. Interestingly, not all compounds featuring a potential chelation pocket exhibited type V interactions, with the notable example being rutaecarpine, which instead was stabilized by type II interactions for all its sites (Figure S24). This result indicated that a guest molecule needed to have an appropriate size and shape for it to be able to approach the Co^2+^ dimer, which is reminiscent of a substrate binding to active sites in enzymes. This adaptable binding mechanism, utilizing coordination bonding that responds to guest characteristics, represents a defining feature of APF-80. It is important to note that additional hydrogen bonds were often observed for all of the interaction types except type I, either with the HAP core or with the interstitial water molecules, which provided the necessary directional interactions helping to align the guests.

**Figure 9:**
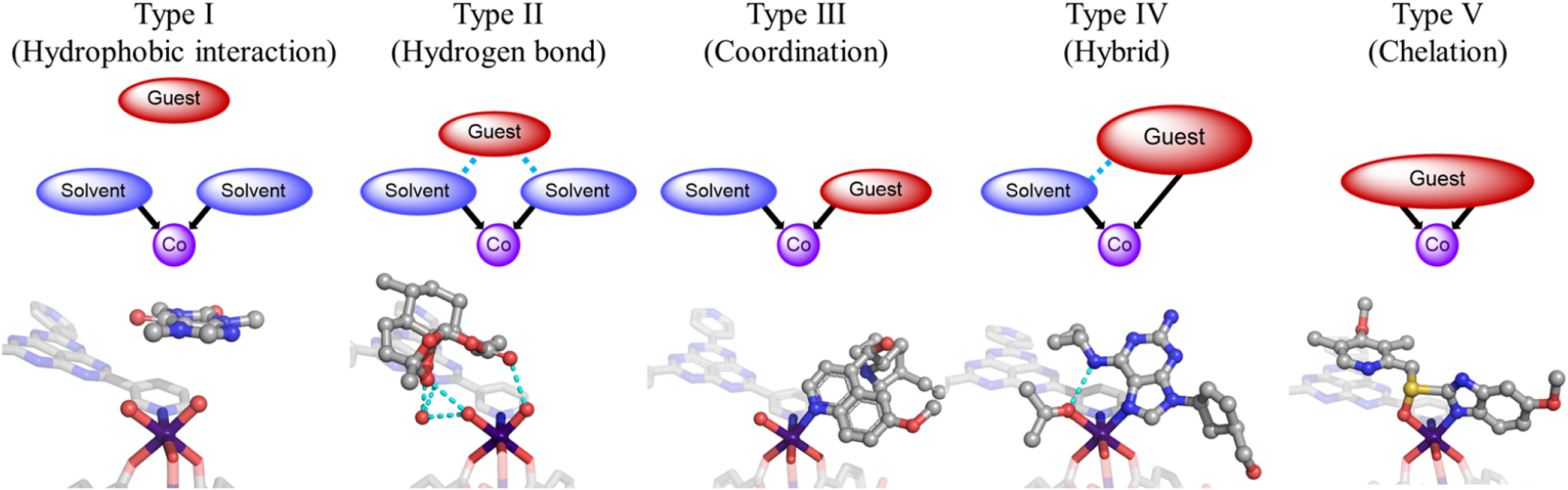
Simplified models of five types of interactions observed between guests and APF-80 (*upper*). Select examples of encapsulated guest structures highlighting each corresponding interaction type: (I) caffeine, (II) artemisinin, (III) quinine, (IV) abacavir, and (V) omeprazole (*lower*). Cyan dash lines represent hydrogen bond contacts. Co, purple; C, gray; O, red; N, blue; and S, yellow. Hydrogen atoms were omitted for clarity.

In previous crystalline sponge reports, guests were typically immobilized inside the pores using a limited range of interactions. For example, in the frameworks that rely on the coordinative alignment strategy, only single-point coordination interactions between the guest and the host were observed, which significantly narrowed the scope of analyzable compounds. In contrast, APF-80 exhibited a diverse range of interaction modes with different guest sites, including coordination to Co^2+^ centers, hydrogen bonding with water molecules and the HAP core, and hydrophobic interactions. The availability of multiple complementary interactions ensured that at least one highly ordered atomically resolved site could be observed for each encapsulated compound. Guest encapsulation induced distinctive framework distortions, with the orthorhombic unit cell undergoing deformation along its axes, which was dependent on the guest molecular geometry and involved interactions (Table S4). The formation of specific interactions between the encapsulated guests and the framework components induced local structural distortions, which in turn led to more significant global changes to the unit cell of APF-80. The mechanism underpinning the adaptable behavior of APF-80 was denoted as multimodal synergistic alignment (MSA). The framework pore could provide a more favorable arrangement of interaction points for the effective capture of various functional groups in the target molecules, allowing it to crystallographically resolve a broader range of guest structures.

### Calculations

In previous reports, density functional theory (DFT) level calculations (DFT-2) were employed to analyze crystalline sponge structures, which uncovered a correlation between the guest binding energy and temperature factors (B factor).^80^ These results demonstrated that strong host–guest interactions suppressed the vibrational and rotational motion of encapsulated molecules. In some instances, contributions from unmodeled solvents or intermolecular interactions led to higher-temperature factors, despite weaker binding energies. In another study, conformational energies of guest molecules inside crystalline sponges were examined and compared to gas phase and pure/cocrystal solid structures.^81^ It was revealed that the encapsulated states displayed reduced molecular strain and closely resembled the optimized global minimum gas-phase structures rather than the molecules packed in a solid state.

The existence of multiple well-resolved guest sites inside APF-80, each classified based on their interaction modes, presented an opportunity to assess the relative stabilization energy contributions. The obtained encapsulated host–guest structures were modeled computationally using the XTB2 method to determine the site-dependent binding energies. The crystal structures determined by X-ray diffraction were used directly as the initial structures without any optimization. The site binding energy (*E*_binding_) was calculated by subtracting the total energy of the empty MOF (*E*_MOF_) and the potential energy of the guest (*E*_guest_) from the energy of the MOF containing the corresponding guest site (*E*_MOF+guest_) (Table S5). The analysis revealed a positive correlation between *E*_binding_ and guest occupancy, with a correlation coefficient of 0.561, which is consistent with the preferential guest adsorption into the more stabilized locations inside the pore (Figure 10). The binding energy ranges depending on the interaction types were 17.8–19.3 kJ mol^–1^ (type I), 66.0–302 kJ mol^–1^ (type II), 141–255 kJ mol^–1^ (type III), 258–292 kJ mol^–1^ (type IV), and 322–392 kJ mol^–1^ (type V). This trend confirmed that the increase in contributions from stronger interactions, specifically hydrogen bonding and metal coordination, resulted in higher site energy. While the XTB method did not yield highly quantitative binding energies, the deviations in the values of the outliers from a linear correlation with the occupancy (by hundreds of kJ/mol in some cases) far exceeded the typical uncertainty of XTB for noncovalent interaction energies (at most up to a few tens of kJ/mol). There were several important contributing factors not accounted for in the binding energy calculations that could have a strong influence on the correlation coefficient. First, the diffusion of molecules throughout the pores of APF-80 was not modeled. To reach their preferred sites, guest molecules must pass through small apertures of 4.36 Å in size. If the molecular size and shape do not match these openings, then the diffusion processes can induce a significant energy barrier limiting the guest occupancy in the final structure. Furthermore, in the host–guest structures featuring strong binding energies, the trapping of molecules at the crystal surface sites could occur, which would prevent their diffusion into the bulk crystal. Second, the solvent molecules occupying the framework pores prior to encapsulation were excluded from the *E*_MOF+guest_ calculations. These molecules needed to be displaced by the guests, thus incurring an energy penalty, which was not accounted for. Third, there were low-occupancy disordered solvent molecules present in the host–guest structures after encapsulation, which could not be properly modeled crystallographically. As a result, the influence of the unmodeled solvents on the guest sites could not be included in the binding energy calculations. Despite several unaccounted factors, the interrelationship between occupancy obtained from X-ray crystallography and the calculated *E*_binding_ values was clearly established. Further refinements of the calculation methodology will be necessary to improve the correlation coefficient. These findings could help us to better understand the factors that govern the encapsulation and molecular alignment processes that facilitate a successful crystallographic visualization, which could then be applied to the design of new crystalline sponges.

**Figure 10:**
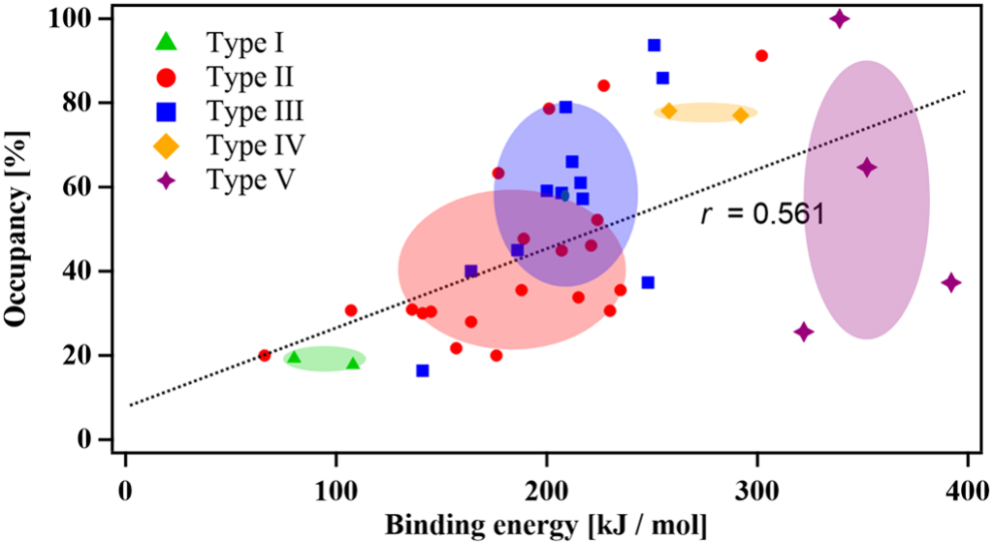
Plot of *E*_binding_ vs crystallographic occupancy divided based on interaction types of guests encapsulated inside APF-80. The colored ellipses represent approximate areas with a high likelihood of finding the corresponding interaction type.

## Conclusions

In this study, a new mixed pyridyl-substituted hexaazaphenalene-based ligand (referred to as 344-TPHAP or **344-L**) was successfully prepared using a stepwise HAP condensation procedure. It was then used to assemble a novel MOF, denoted as APF-80, together with Co^2+^ ions and 1,4-benzenedicarboxylate coligands. The framework incorporated water-mediated hydrogen-bonding networks, which were anchored at chelating pockets formed between the HAP core and coordinated water molecules. Moreover, one of the Co^2+^ centers contained two solvent-occupied sites in a *cis* configuration, which could be displaced by other coordinating groups. To take advantage of these structural motifs, APF-80 was tested as a crystalline sponge to capture and elucidate the structures of pharmaceutically active alkaloid compounds featuring nucleophilic groups. These kinds of molecules are especially challenging to analyze using the crystalline method because of their tendency to damage the host structures. Encapsulation of 12 distinct compounds was performed, and their structures were successfully resolved including one previously unreported intermediate. The framework crystals were soaked in the corresponding guest solutions at 40 °C without extensive condition optimization. Guest molecules were precisely immobilized inside the pores via a combination of coordination and hydrogen bonds, resulting in high-quality crystallographic data, which required minimal restraints or constraints to model. Five distinct interaction modes were identified in the resulting structures, which were classified based on the interactions between the encapsulated molecules and the Co^2+^ dimers. The diverse interactivity inside APF-80 facilitated the effective accommodation of a variety of molecular shapes and functional groups, stabilizing their positions and orientations inside the pore. The mechanism responsible for guest capture was dubbed multimodal synergistic alignment (MSA). The site binding energies were systematically evaluated using computational modeling, which revealed a positive correlation between energy values and crystallographic guest occupancy. These results firmly established APF-80 as a versatile crystalline matrix that can successfully analyze nucleophilic compounds without losing its structural integrity. The presented findings make a significant contribution to the advancement of the crystalline sponge methodology and also provide fundamental knowledge about the factors that govern molecular alignment inside porous frameworks.

## Supplementary Material



## Notes

Experimental section: chemicals, general methods, single-crystal X-ray diffraction, binding energy calculations, synthetic procedures and characterization of **344-L** and APF-80, solvent exchange of APF-80, and guest encapsulations in APF-80; crystallographic results: structures of all guest sites, crystallographic restraints and constraints, the lengths of Co–N bonds in quinoline-containing complexes, unit cell volumes of APF-80 after encapsulation, binding energies of each guest site, crystallographic tables, and checkCIF alerts and their responses. (PDF)
