# Supplementary material for: Structural Elucidation of Nucleophilic Compounds through Synergistic Coordination and Hydrogen Bonding in a Metal–Organic Framework
Source: J Am Chem Soc. 2025 Jul 31;147(32):29013–25. doi: 10.1021/jacs.5c07192 (PMC12356596; doi:10.1021/jacs.5c07192)
Supplement: Supplementary file 1 [file ja5c07192_si_001.pdf]

Supporting Information

Structural Elucidation of Nucleophilic Compounds through Synergistic Coordination and Hydrogen Bonding in a Metal-Organic Framework

Tomoki Nakagawa,[1] Yuki Wada,[1,2]* Bun Chan,[3] Taichi Baba,[1] Kengo Hanaya,[4] Yuta Koseki,[5] Ryuji Asano,[5] Katsuyuki Aoki,[5] Pavel M. Usov,[1] and Masaki Kawano[1,2]*

[1]Department of Chemistry, School of Science, Institute of Science Tokyo, 2-12-1 Ookayama, Meguro-ku, Tokyo 152-8550, Japan

[2]TEKMOF Co., Ltd., INDEST, 3-3-6 Shibaura, Minato-ku, Tokyo 108-0023, Japan

[3]Graduate School of Engineering, Nagasaki University, Bunkyo 1-14, Nagasaki-shi, Nagasaki 852-8521, Japan

[4]Faculty of Pharmacy, Keio University, 1-5-30 Shibakoen, Minato-ku, Tokyo 105-8512, Japan

[5]Tsumura Botanical Raw Materials Research Laboratories, Tsumura & Co., 3586, Yoshiwara, Ami-machi, Inashiki-gun, Ibaraki 300-1192, Japan

*Corresponding author. Y. Wada, e-mail: yuki.wada@chem.sci.isct.ac.jp and M. Kawano, e-mail: mkawano@chem.sci.isct.ac.jp

Table of Contents

Experimental section S3

Chemicals........ S3

General methods S3

Single crystal X-ray diffraction........ S3

MOF-guest binding energy calculations S4

Synthesis of 4,6-diamino-2-(3-pyridyl)-5-pyrimidinecarbonitrile (3PP) S5

Scheme S1. Synthesis of 3PP. S5

Synthesis of 2-(3-pyridyl)-5,8-di(4-pyridyl)-1,3,4,6,7,9-hexaazaphenalene (344-TPHAP, **344-L**). S6

Scheme S2. Synthesis of 344-TPHAP (**344-L**)........ S6

Synthesis of APF-80 .... S7

Figure S1. ^{1}H NMR (DMSO-d_6) spectrum of 3PP. S8

Figure S2. ^{13}C NMR (DMSO-d_6) spectrum of 3PP. S8

Figure S3. The crystal structure of 3PP S9

Figure S4. ^{1}H NMR spectrum of TPP(344-TPHAP). S10

Figure S5. ^{13}C NMR (DMSO-d_6) spectrum of TPP(344-TPHAP). S10

Figure S6. Solution state UV-Vis spectra of 3PP and TPP(344-TPHAP). S11

Figure S7. Diffuse reflectance UV-Vis spectra of TPP(344-TPHAP) and APF-80........ S11

Figure S8. The IR spectrum of 3PP. S12

Figure S9. IR spectra of TPP(344-TPHAP) and APF-80. .... S12

Figure S10. IR spectra of TPP(344-TPHAP) and APF-80. .... S13

Figure S11. Powder X-ray diffraction pattern of APF-80. .... S14

Figure S12. Le Bail refinement of the APF-80 powder pattern. S14

Figure S13. TGA and DSC data for APF-80. .... S15

Solvent exchange of APF-80........ S16

Guest molecule encapsulations in APF-80........ S16

Table S1. Encapsulation conditions. S16

Crystallographic results S17

Figure S14. Artemisinin encapsulated structure summary. S17

Figure S15. Caffeine encapsulated structure summary. S18

Figure S16. Caffeine stacking between two BDC ligands in APF-80........ S19

Figure S17. The disorder models of caffeine in APF-80........ S19

Figure S18. Omeprazole encapsulated structure summary. S20

Figure S19. Nicotine encapsulated structure summary. S21

Figure S20. Quinine encapsulated structure summary. S22

Figure S21. Quinidine encapsulated structure summary. S23
Figure S22. Cytisine encapsulated structure summary. S24
Figure S23. Cyclopenthiazide encapsulated structure summary. S25
Figure S24. Rutaecarpine encapsulated structure summary. S26
Figure S25. Voriconazole encapsulated structure summary. S27
Figure S26. Abacavir encapsulated structure summary. S28
Figure S27. Compound-A encapsulated structure summary. S29
Table S2. A list of crystallographic restraints and constraints applied during the structure analysis of encapsulated guests. S30
Figure S28. Visualization of crystallographic restraints and constraints. S32
Table S3. The lengths of Co-N coordination bonds in previously reported quinoline containing complexes. S34
Table S4. Unit cell volumes of guest-encapsulated APF-80 structures. S35
Table S5. Calculated binding energies and crystallographic occupancies for each guest site. S36
Table S6. Crystallographic tables. S37
A- or B-level checkCIF alerts and their responses. S44
References S52

Experimental section

Chemicals

Solvents and reagents were purchased from KANTO CHEMICAL CO., INC., TCI Co., Ltd. and FUJIFILM Wako Pure Chemical Corporation, and used without further purification except where noted.

General methods

^{1}H and ^{13}C NMR spectra were collected on a JEOL JNM-ECA400 II instrument. UV-Vis spectra were collected on a JASCO V-770 spectrophotometer. For solution-state measurements, quartz cell (optical path length = 1 cm) was used. The solid-state measurements were performed in the diffuse reflectance mode. The samples were mixed with $BaSO_4$, which was used as a background. Thermogravimetric analysis differential scanning calorimetry (TGA-DSC) was measured using Simultaneous Thermogravimetry STA449F3 Jupiter analyzer NETZSCH. The powdered samples were loaded into Al_2O_3 pans and heated at 5 K/min rate under a flow of N_2. Elemental analyses were measured using JScience JM-10 instrument. Fourier transform infrared (FTIR) spectra were measured on a Nicolet iS5 spectrometer from Thermo Fisher Scientific using attenuated total reflectance (ATR) method. Rigaku SmartLab diffractometer employing Ni-filtered Cu Kα (λ = 1.54184 Å) line focused radiation at 2000 W (40 kV, 50 mA) power was used for high resolution PXRD measurements. The sample suspended in DMA was loaded into capillary tubes (borosilicate glass, inner diameter = 0.5 mm, outer diameter = 0.8 mm), which were flame sealed to prevent solvent evaporation. The patterns were collected using 1° min^{-1} scan speed and 120 rpm rotation rate for the capillary. Le Bail refinement was conducted using Rietica.

Single crystal X-ray diffraction

The diffraction data for 3PP was recorded on a Rigaku Varimax diffractometer with Saturn system equipped with a Rigaku GNNP low temperature device using graphite-monochromated Mo Kα radiation (λ = 0.71075 Å). For all other crystals, the X-ray analysis was performed on a diffractometer equipped on a synchrotron beamline BL-5A at KEK (the High Energy Accelerator Research Organization, Japan) with a Pilatus3 S6M detector (λ = 0.7500 Å, T = 95 K). XDS[1] software was used for the processing and data reduction. The structures were solved by Dual space methods (SHELXT-2018[2]) and refined by full-matrix least squares calculations on F^2 (SHELXL-2018[3]) using the OLEX2[4] program package. All non-hydrogen atoms were refined with anisotropic displacement parameters. All hydrogen atoms were created with ideal geometry and refined using a riding model.

MOF-guest binding energy calculations

First, to create initial files for calculation, a set of cif files for each crystallographic site was created, and the water molecules interacting with the guest were identified and the other pore contents were deleted. The interaction energy between the MOF and each guest site was calculated using the XTB method with the SCM DFTB program. The site binding energy (E_{binding}) was calculated by subtracting the total energy of the empty MOF (E_{MOF}) and the potential energy of the guest (E_{guest}) from the energy of the MOF containing the corresponding guest site ($E_{\mathrm{MOF+guest}}$).

Synthesis of 4,6-diamino-2-(3-pyridyl)-5-pyrimidinecarbonitrile (3PP)

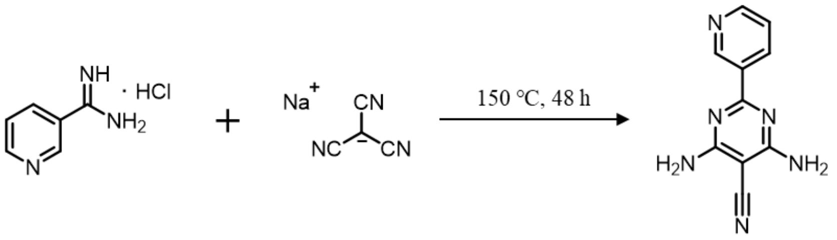

Scheme S1. Synthesis of 3PP.

3-Amidinopyridine hydrochloride (3.0 g, 19 mmol) and sodium tricyanomethanide (2.0 g, 18 mmol) were ground together using mortar and pestle and then placed in a Teflon-lined stainless-steel autoclave, which was heated at 150 °C for 2 days. After colling down, the resultant black solid was ground into a fine powder, which was loaded into a Soxhlet extractor, and then extracted with MeOH for 1 day. The obtained solution was reduced to dryness, and the resultant brown solid was redissolved in 3 L of ethyl acetate. The solution was stirred for 1 h while heating at 70 °C, and then filtered. The filtrate was loaded into a silica gel column and the main fraction was eluted with ethyl acetate. The resultant solution was evaporated to give a pale-yellow solid, which was resuspended in 50 ml of water. Filtration of the suspension gave the desired product as a pale-yellow powder which was dried under vacuum (543 mg, 14%). ^{1}H NMR (400 MHz, DMSO-d_6): δ = 7.34 (br, 4H), 7.50 (ddd, 1H, J = 0.9, 4.8, 8.0 Hz), 8.48 (ddd, 1H, J = 2.1, 2.1, 7.9 Hz), 8.67 (dd, 1H, J = 1.8, 4.6 Hz), 9.36 (dd, 1H, J = 0.9, 2.3 Hz). ^{13}C NMR (100 MHz, DMSO-d_6): δ = 66.06, 115.86, 123.37, 132.59, 135.23, 149.30, 151.35, 162.70, 164.75. Elemental analysis calcd. (%) for $C_{10.00}H_{8.04}N_{6.00}O_{0.02}$ (= $(C_{10}H_8N_6)\cdot(H_2O)_{0.02}$): C, 56.50; H, 3.81; N, 39.53. Found: C, 56.52; H, 3.87; N, 39.53. After the completion of the solid-state reaction colorless crystals were found on the wall of the Teflon vessel, which were suitable for single-crystal X-ray diffraction (SCXRD) analysis. The obtained crystal structure confirmed the formation of 3PP (Figure S3).

Synthesis of 2-(3-pyridyl)-5,8-di(4-pyridyl)-1,3,4,6,7,9-hexaazaphenalene (344-TPHAP, **344-L**).

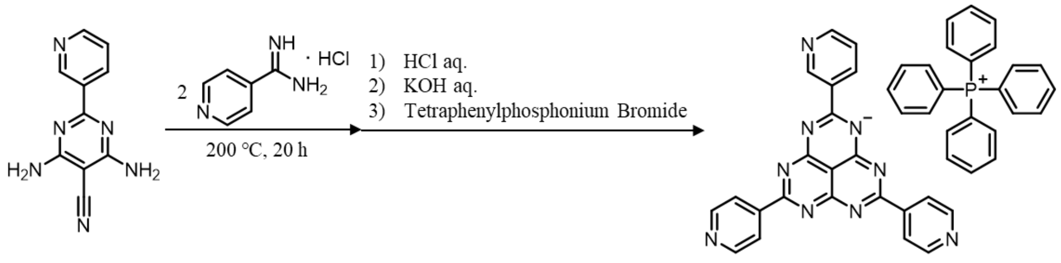

Scheme S2. Synthesis of 344-TPHAP (**344-L**).

4-Amidinopyridine hydrochloride (945.6 mg, 6 mmol) and 3PP (424.4 mg, 2 mmol) were ground together and the solid-state mixture was dried under vacuum at 60 °C for 3 h. It was then placed into a Teflon-lined stainless-steel autoclave and sealed under N_2 atmosphere to avoid moisture. The reaction vessel was heated at 200 °C for 20 h. After cooling down, the resultant brown solid was dissolved in 5 mL of 5 M aqueous HCl solution. Addition of 200 mL of acetone to the solution caused precipitation of a sticky brown solid. The cloudy white suspension was removed by decantation. The remaining brown solid was redissolved in 10 mL of water, and the solution was added dropwise into 50 mL of 5 M aqueous KOH solution with rapid stirring causing precipitation of a brown solid, which was isolated by vacuum filtration. It was redissolved in 50 mL of water while heating at *ca.* 80 °C, and the resultant solution was adjusted to pH 8-10 using 5 M aqueous HCl solution. It was added dropwise into 50 mL of aqueous tetraphenylphosphonium bromide (838.6 mg, 2 mmol) solution while heating at *ca.* 80 °C. A brown oil appeared and settled at the bottom of the flask, and the supernatant was carefully removed using a pipette. The crude compound was purified by column chromatography using silica gel (first 1:1 ethyl acetate/CH_2Cl_2, then 5:5:1:2 ethyl acetate/CH_2Cl_2/triethylamine/MeOH). The product fractions were evaporated to give the pure TPP(344-TPHAP) as a yellow oil, which was further dried under vacuum at 60 °C turning into a yellow solid (168 mg, 24%). ^{1}H NMR (400 MHz, DMSO-d_6): δ= 7.57 (ddd, 1H, J = 0.9, 4.8, 8.0 Hz), 7.74 (m, 8H), 7.81 (m, 8H), 7.97 (m, 4H), 8.39 (dd, 4H, J = 1.8, 4.6 Hz), 8.72 (dd, 1H, J = 1.8, 4.6 Hz), 8.77 (dd, 4H, J = 1.8, 4.6 Hz), 8.81 (ddd, 1H, J = 2.1, 2.1, 7.9 Hz), 9.64 (dd, 1H, J = 1.4, 2.3 Hz). ^{13}C NMR (100 MHz, DMSO-d_6): δ = 103.45, 117.24, 118.13, 122.24, 123,48, 130.44, 134.37, 134.56, 135.33, 135.68, 146.38, 149.60, 150.10, 151.07, 165.93, 166.66, 166.71. Elemental analysis calcd. (%) for $C_{49.54}H_{51.68}PN_{9.00}O_{6.30}$ (= $(C_{24}H_{20}P)(C_{22}H_{12}N_9)\cdot(CH_3OH)_{3.54}\cdot(H_2O)_{2.76}$): C, 65.75; H, 5.76; N, 13.93. Found: C, 65.75; H, 6.05; N, 13.93.

Synthesis of APF-80

$CoBr_2$ (4.4 mg, 20 μmol), TPP(344-TPHAP) (7.4 mg, 10 μmol) and 1,4-benzenedicarboxylic acid (BDC) (2.5 mg, 15 μmol) were dissolved in *N,N'*-dimethylacetamide (DMA) (1 mL). The reaction mixture was placed in a 2 mL glass vial and heated in a heating block at 100 °C for 2 days. After cooling down to room temperature, the reaction solution was decanted leaving magenta-colored crystals behind (0.2 mg, 2%). Fresh DMA (1 mL) was added to the vial, and the solution was decanted again. The process was repeated three times. The framework structure was solved using SCXRD. The bulk purity was confirmed by powder X-ray diffraction measured in DMA. The crystals were stored in DMA for further solvent exchange, whereas for the elemental analysis, UV-vis spectroscopy, IR spectroscopy and TGA-DSC, they were collected by filtration and dried. Elemental analysis calcd. (%) for $Co_4C_{105.56}H_{145.37}N_{27.39}O_{33.82}$ (= $Co_4(C_{22}H_{12}N_9)_2(C_8H_4O_4)_3(C_4H_9NO)_4{\cdot}(C_4H_9NO)_{5.39}{\cdot}(H_2O)_{12.43}$): C, 49.24; H, 5.69; N, 14.90. Found: C, 49.24; H, 5.69; N, 14.91.

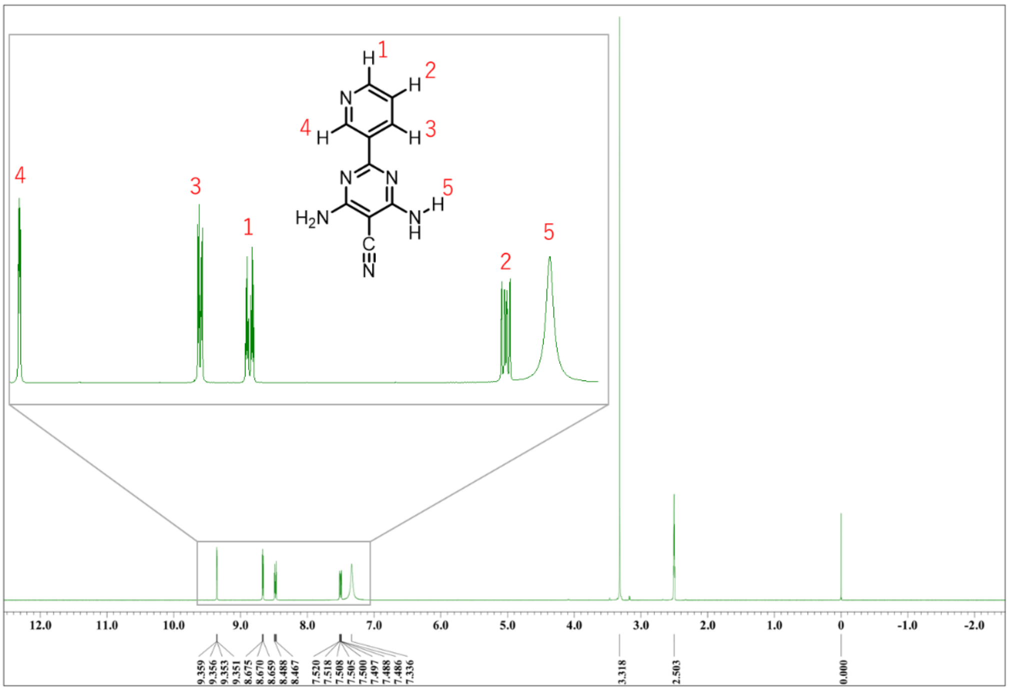

Figure S1. ^{1}H NMR (DMSO-d_6) spectrum of 3PP.

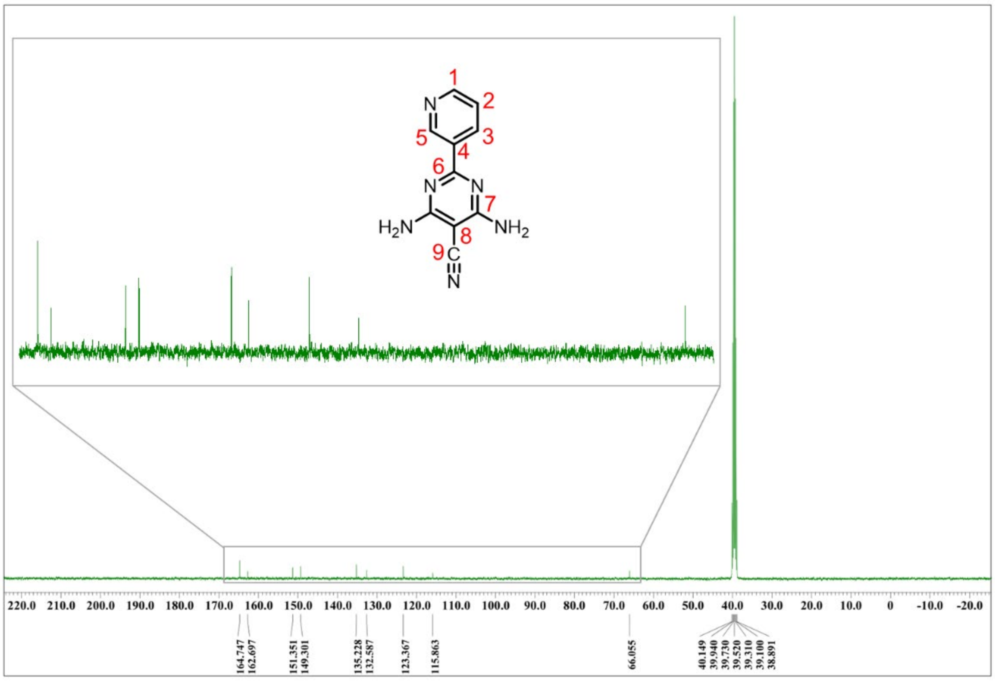

Figure S2. ^{13}C NMR (DMSO-d_6) spectrum of 3PP.

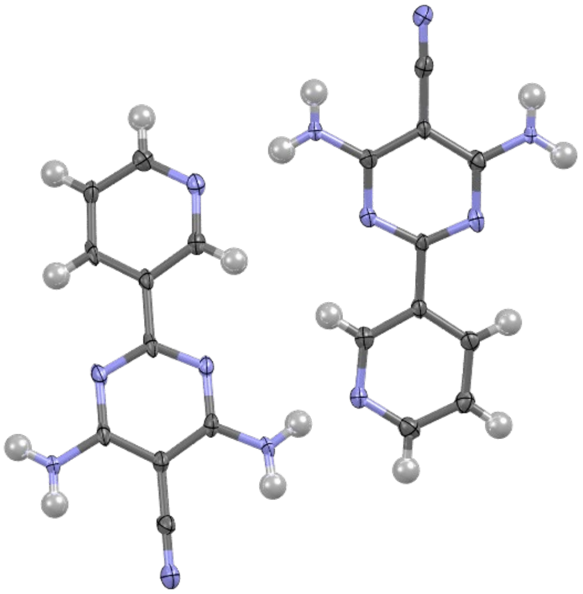

Figure S3. The single crystal structure of 3PP shown as an ORTEP diagram with 50% probability. C – grey, H – white and N – blue.

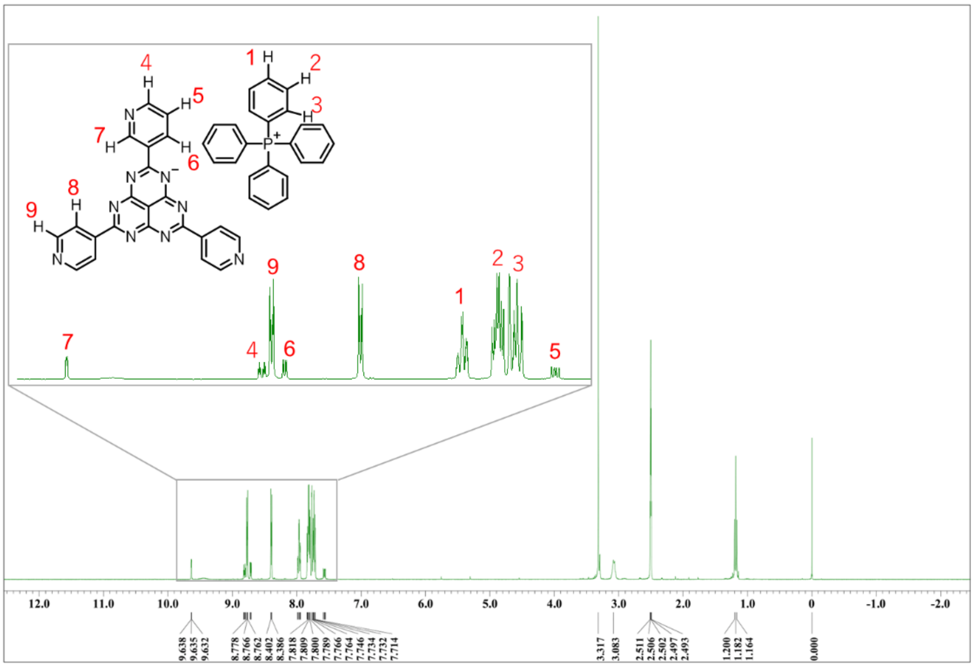

Figure S4. ^{1}H NMR (DMSO-d_6) spectrum of TPP(344-TPHAP).

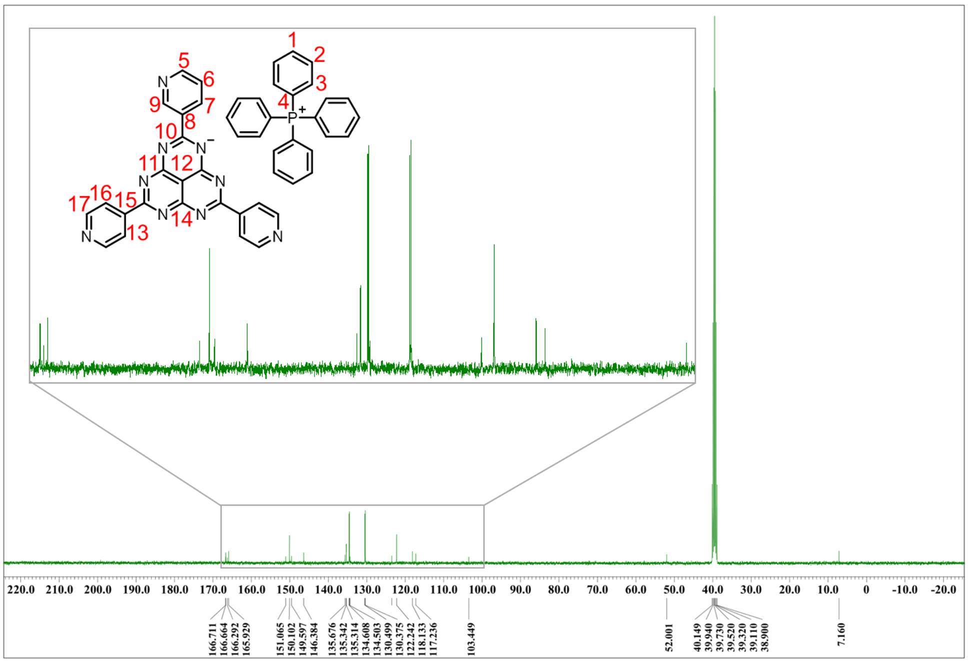

Figure S5. ^{13}C NMR (DMSO-d_6) spectrum of TPP(344-TPHAP).

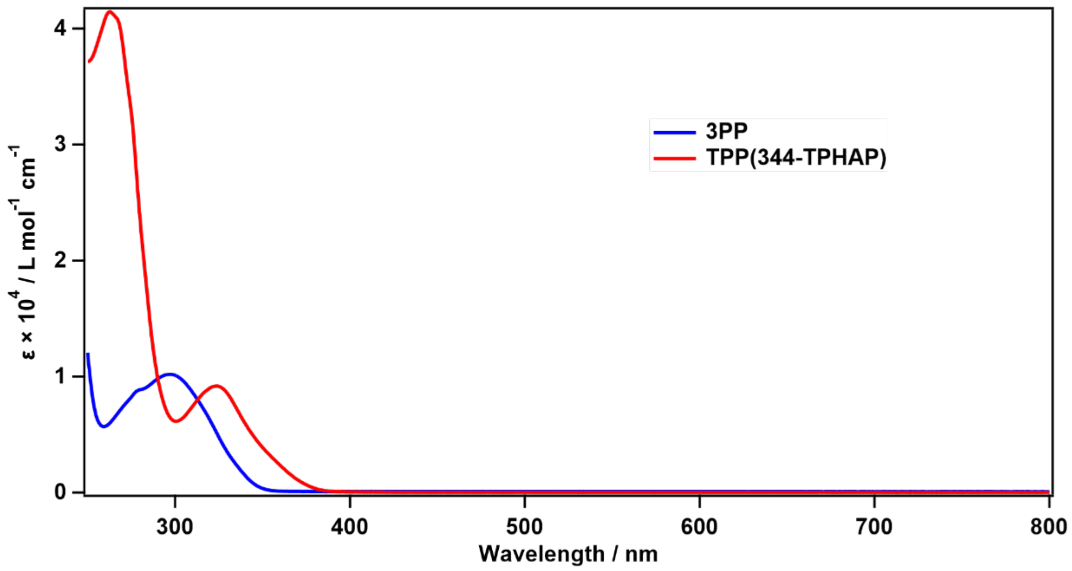

Figure S6. Solution-state UV-Vis spectra of 3PP and TPP(344-TPHAP) measured in MeOH solutions (0.05 mM and 0.03 mM, respectively).

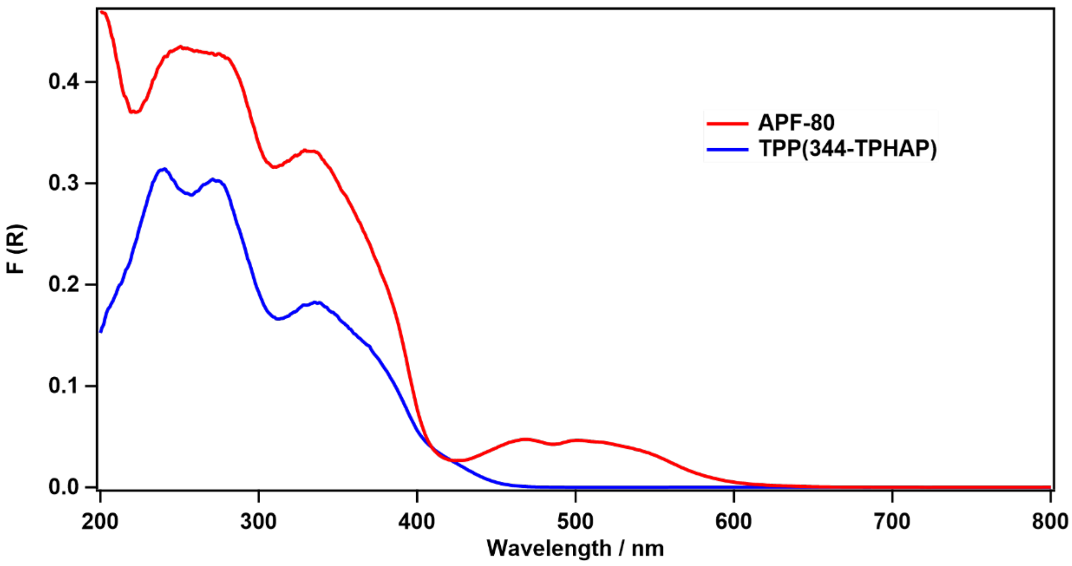

Figure S7. Diffuse reflectance UV-Vis spectra of TPP(344-TPHAP) and APF-80.

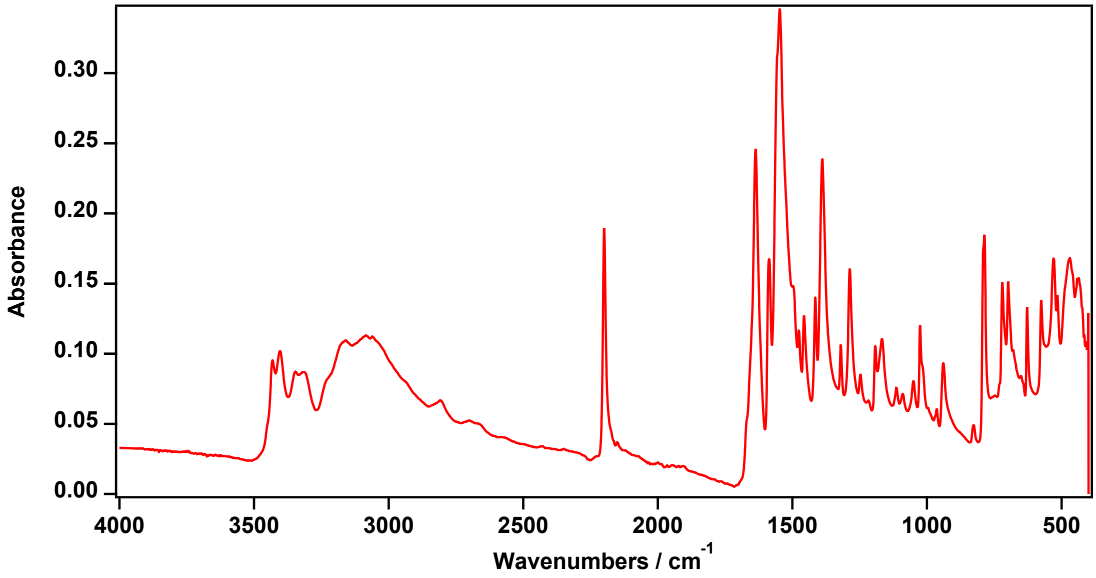

Figure S8. The IR spectrum of 3PP.

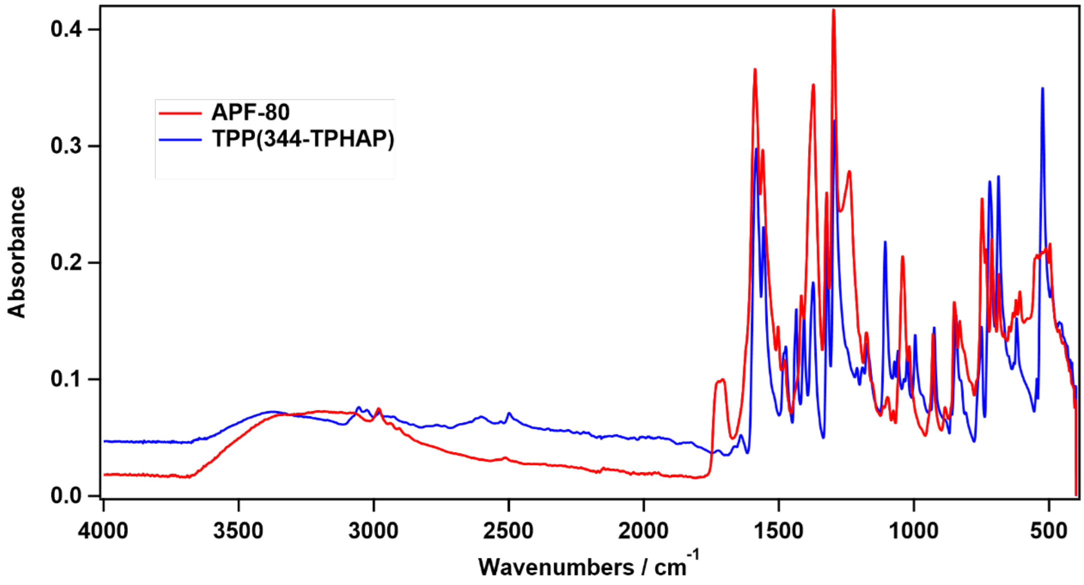

Figure S9. IR spectra of TPP(344-TPHAP) and APF-80.

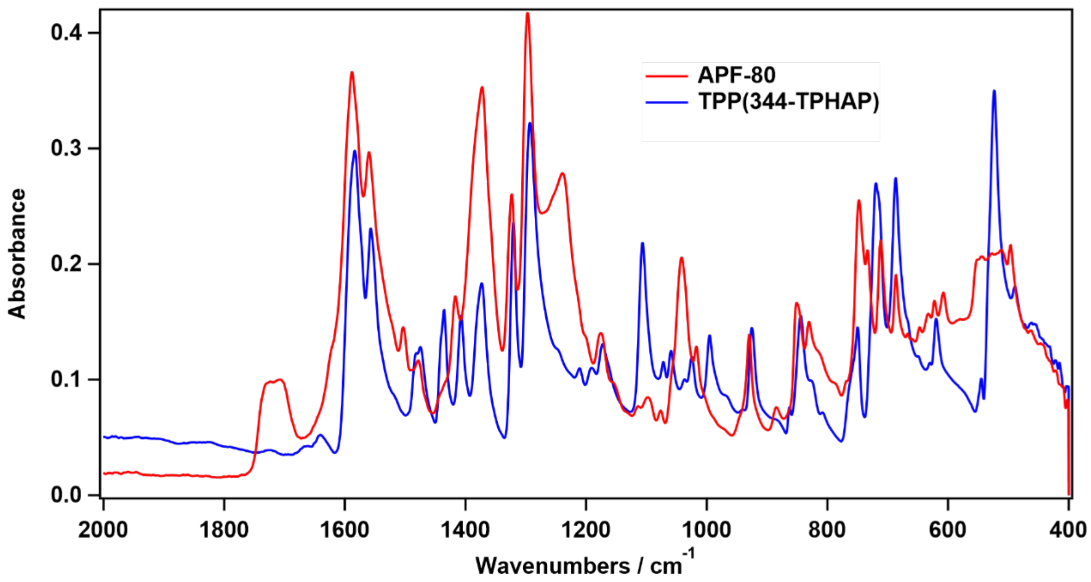

Figure S10. IR spectra of TPP(344-TPHAP) and APF-80 showing the 2000 – 400 cm^{-1} region.

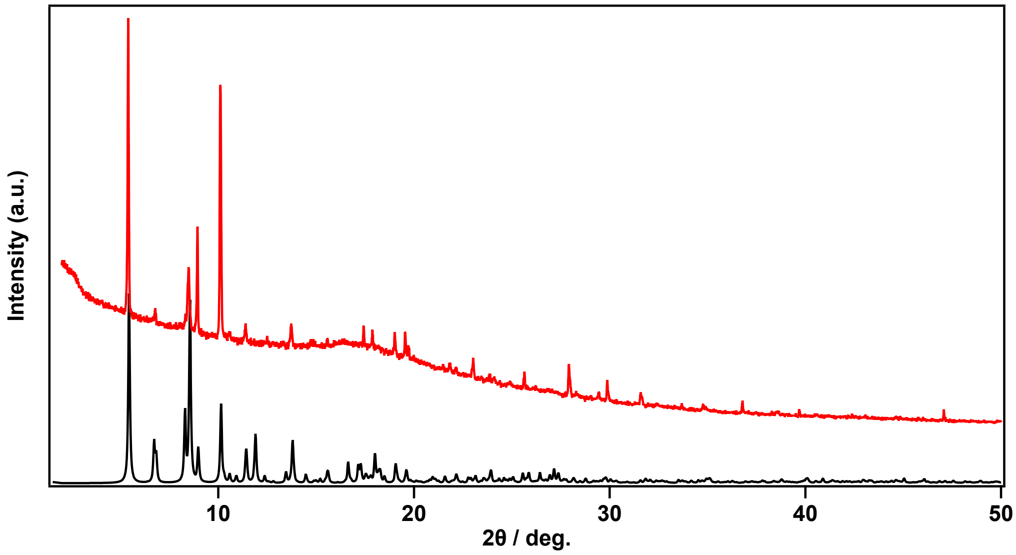

Figure S11. Powder X-ray diffraction pattern of APF-80 measured in DMA (*red*) compared to the simulated pattern from the single crystal structure (*black*).

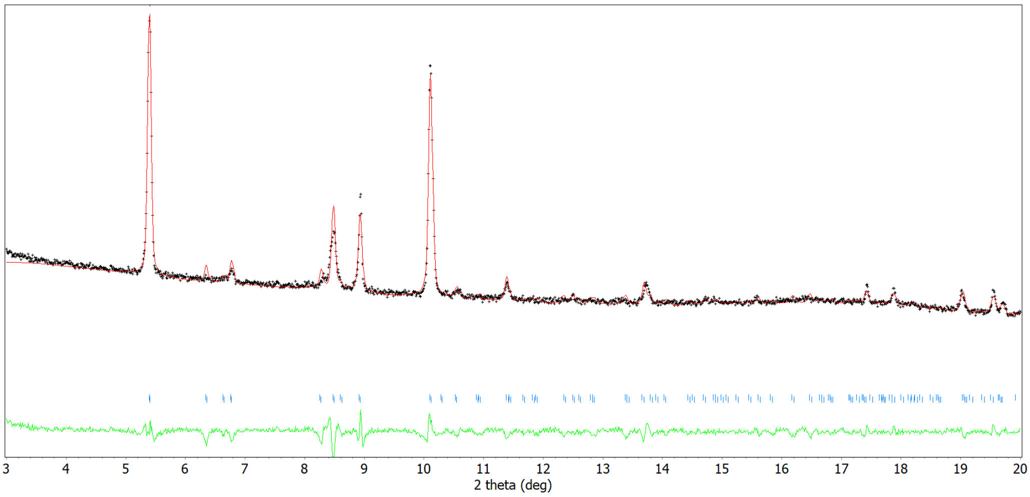

Figure S12. Le Bail refinement of the APF-80 powder pattern measured in DMA. The measured pattern (*black crosses*), Le Bail fit (*red solid line*), residual (*green solid line*) and allowed reflections for the *Pbca* space group at Cu Kα_1 and Kα_2 radiation wavelengths (*blue tick marks*). Fitted unit cell parameters: a = 26.2(4) Å, b = 16.6(4) Å, c = 32.0(8) Å.

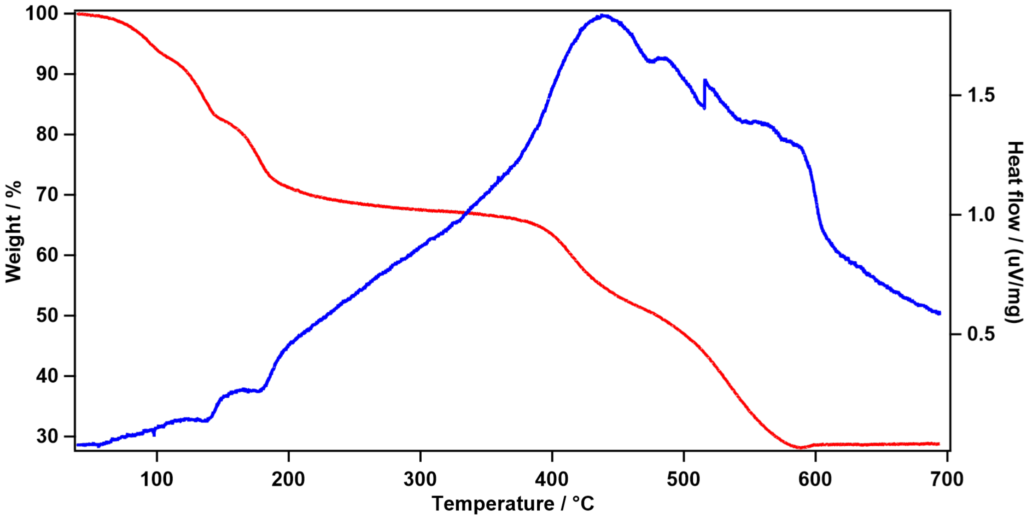

Figure S13. TGA (*red*) and DSC (*blue*) data for APF-80.

Solvent exchange of APF-80

APF-80 crystals were transferred to a 50 mL glass vial and ethyl acetate (40 mL) was added. The solution was decantated and fresh ethyl acetate (40 mL) was added again. This process was repeated three times, and then solution containing APF-80 crystals was kept at 40 °C for 1 day. After that, the solution was replaced with fresh ethyl acetate (40 mL) again. This process was also repeated three times. Finally, the solvent-exchanged APF-80 was stored in ethyl acetate prior to guest encapsulation.

Guest molecule encapsulations in APF-80

The solvent-exchanged APF-80 crystals were transferred to a 2 mL glass vial containing a solvent and a guest for encapsulation (Table S1). The vials were tightly capped and placed in an incubator at 40 °C. After 3 days, the guest loaded crystals were analyzed by SCXRD without any additional treatment.

Table S1. Encapsulation conditions.

Guest name	Guest amount	Encapsulation solvent
Artemisinin	2.1 mg	*n*-heptane 1 mL
Quinine	0.8 mg	*n*-heptane 1 mL
Quinidine	0.8 mg	*n*-heptane 1 mL
Caffeine	1.1 mg	*n*-heptane 0.9 mL + acetone 0.1 mL
Omeprazole	1.2 mg	*n*-heptane 0.9 mL + acetone 0.1 mL
Nicotine	1 μL	*n*-heptane 1 mL
Cytisine	1.4 mg	*n*-heptane 2 mL
Voriconazole	1.2 mg	*n*-heptane 0.9 mL + acetone 0.1 mL
Cyclopenthiazide	1.0 mg	*n*-heptane 0.9 mL + acetone 0.1 mL
Rutaecarpine	1.0 mg	*n*-heptane 1 mL
Abacavir	0.7 mg	*n*-heptane 1.8 mL + acetone 0.2 mL
Compound-A	1.2 mg	*n*-heptane 1.8 mL + acetone 0.2 mL

Crystallographic results

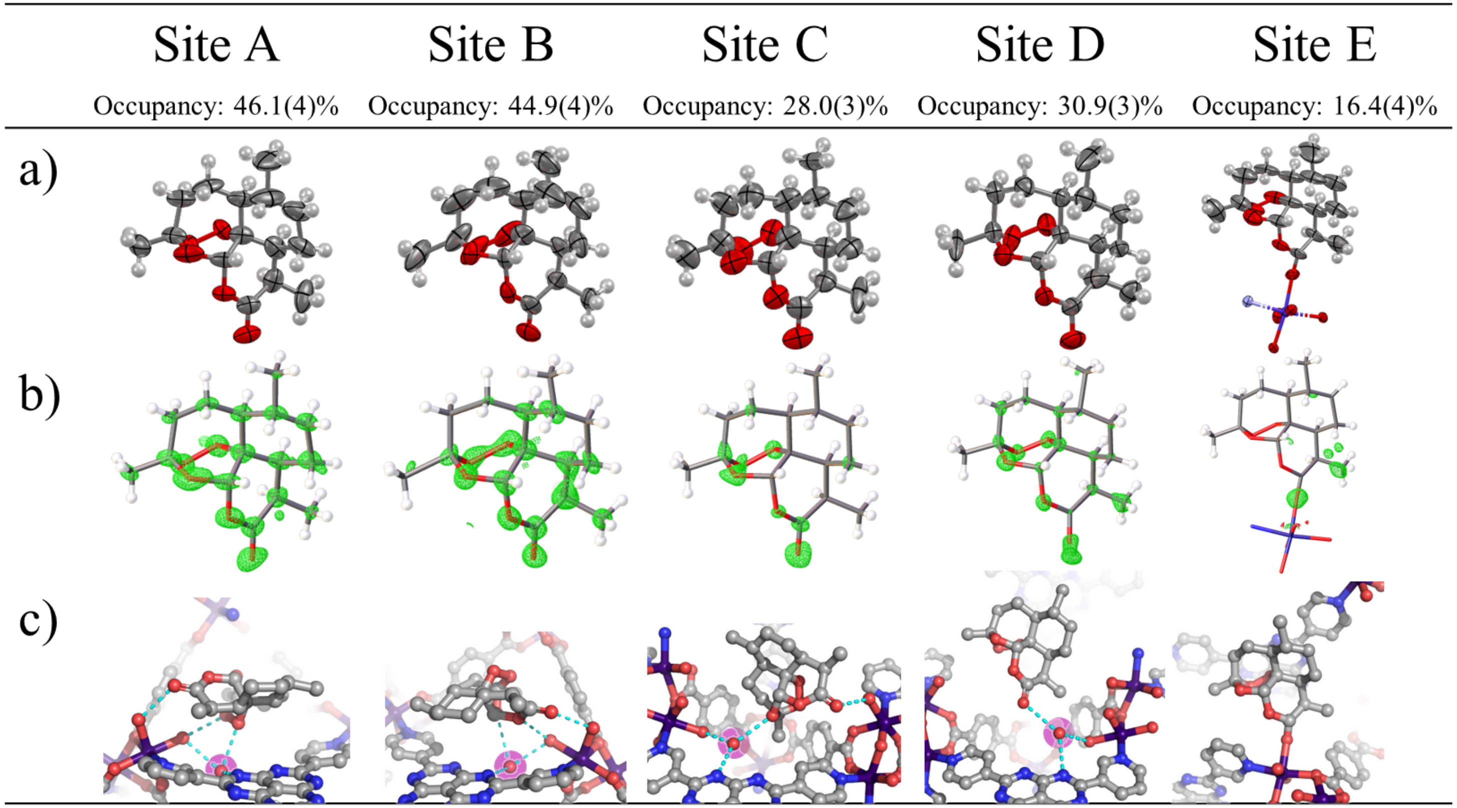

Figure S14. Artemisinin encapsulated structure summary. a) ORTEP diagrams with 50% probability, b) 2Fo-Fc electron density maps (the map threshold: 1.58 e/Å^3), c) the interactions between Co^{2+}, 344-TPHAP, water and artemisinin. Hydrogen atoms were omitted for clarity. The water sites chelated between the HAP core and a water molecule coordinated to Co^{2+} were highlighted in purple. Cyan dash lines represent hydrogen bond contacts. Atom coloring scheme: Co – purple, C – grey, H – white, O – red and N – blue.

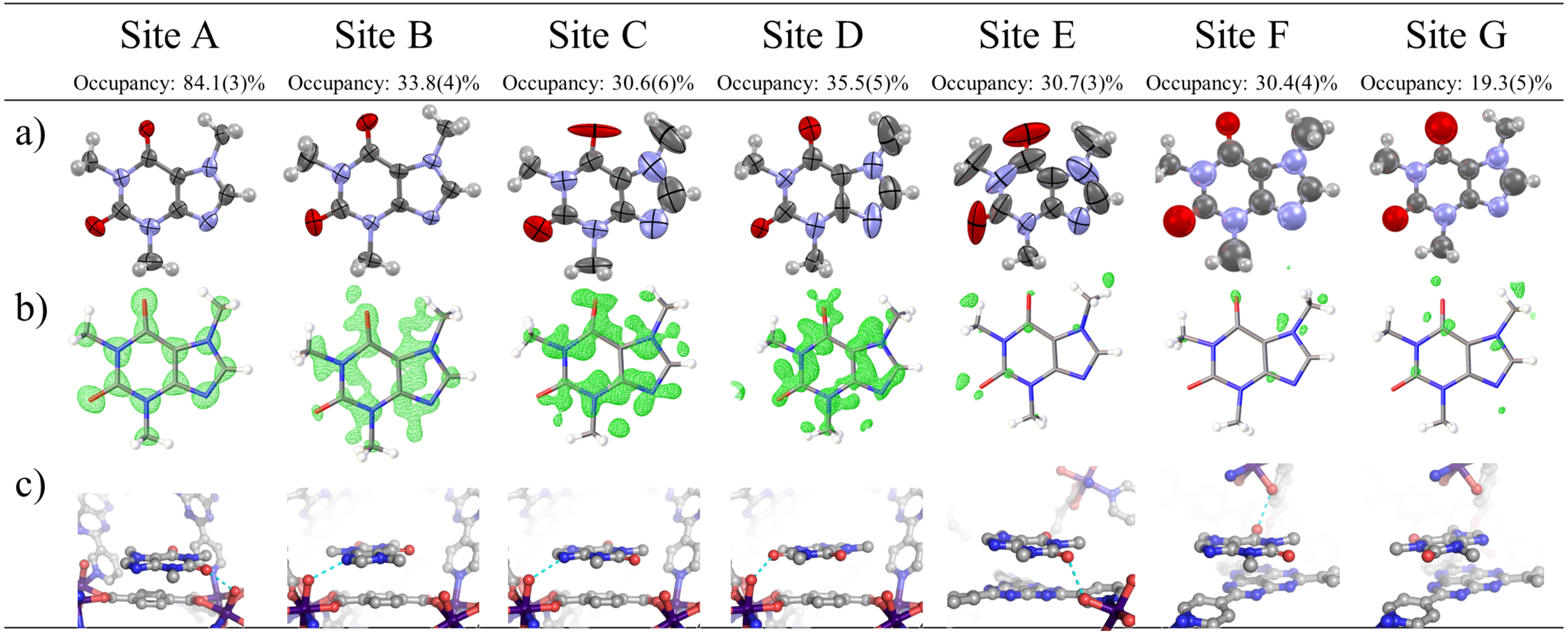

Figure S15. Caffeine encapsulated structure summary. a) ORTEP diagrams with 50% probability, b) 2Fo-Fc electron density maps (the map threshold: 2.0 e/Å^3), c) the interactions between Co^{2+}, 344-TPHAP, water and caffeine. Hydrogen atoms were omitted for clarity. Cyan dash lines represent hydrogen bond contacts. Atom coloring scheme: Co – purple, C – grey, H – white, O – red and N – blue.

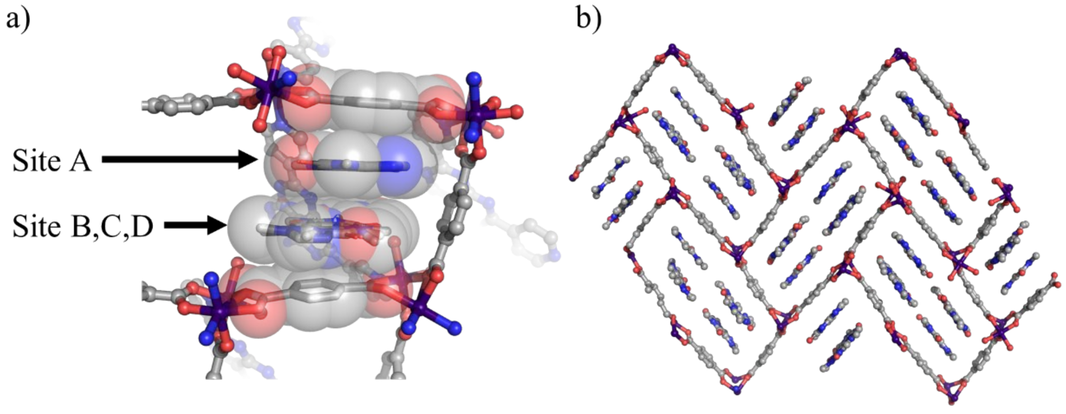

Figure S16. Caffeine stacking between two BDC ligands in APF-80. a) The space-filling models with van der Waals radii applied to BDC and caffeine highlighting the π-π stacking, b) the arrangement of caffeine molecules (sites A, B, C, and D) inside APF-80. Atom coloring scheme: Co – purple, C – grey, O – red and N – blue. Hydrogen atoms were omitted for clarity.

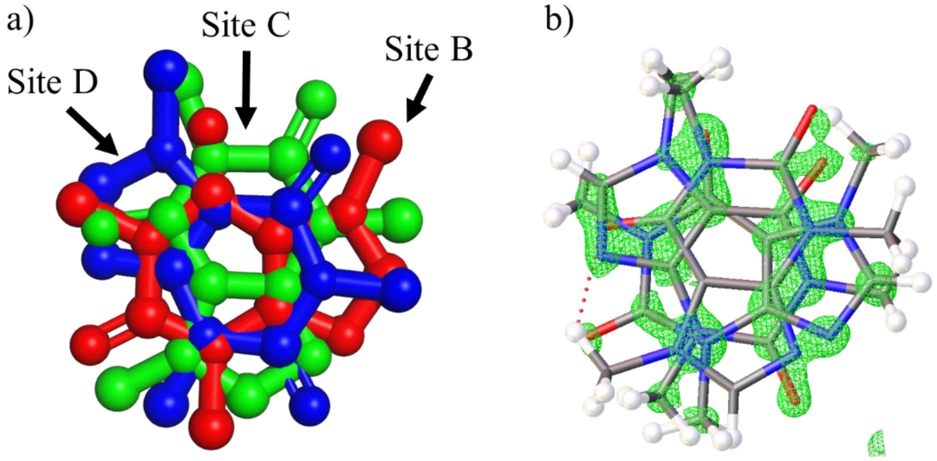

Figure S17. The disorder models of caffeine in APF-80 (site B occupancy = 33.8%, site C occupancy = 30.6%, and site D occupancy = 35.5%). a) Ball and stick model: site B (*red*), site C (*green*) and site D (*blue*). b) The overlapping 2Fo-Fc electron density map for the disorder model (map threshold: 2 e/Å^3). Atom coloring scheme: C – grey, H – white, O – red and N – blue.

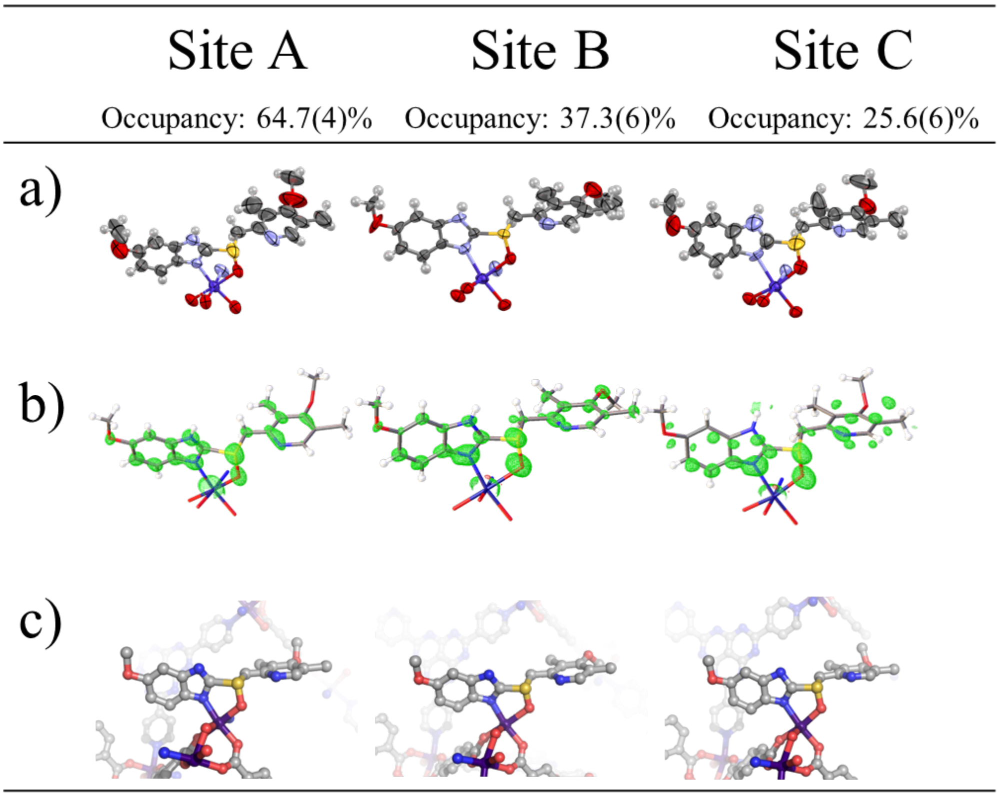

Figure S18. Omeprazole encapsulated structure summary. a) ORTEP diagrams with 50% probability, b) 2Fo-Fc electron density maps (the map threshold: 1.3 e/Å^3), c) the interactions between Co^{2+}, 344-TPHAP, water and omeprazole. Hydrogen atoms were omitted for clarity. Atom coloring scheme: Co – purple, C – grey, H – white, O – red, N – blue and S – yellow.

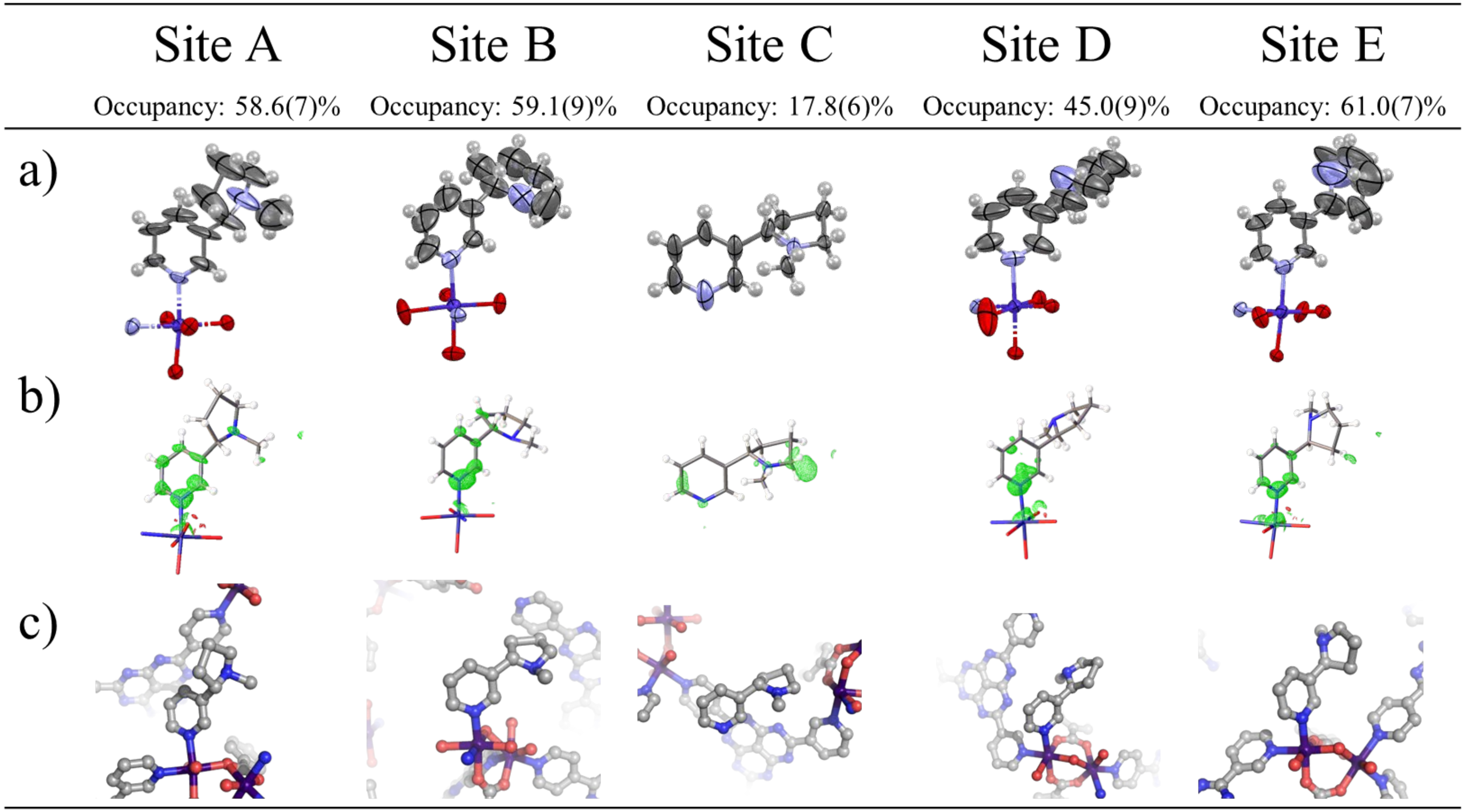

Figure S19. Nicotine encapsulated structure summary. a) ORTEP diagrams with 50% probability, b) 2Fo-Fc electron density maps (the map threshold: 1.6 e/Å^3), c) the interactions between Co^{2+}, 344-TPHAP, water and nicotine. Hydrogen atoms were omitted for clarity. Atom coloring scheme: Co – purple, C – grey, H – white, O – red and N – blue.

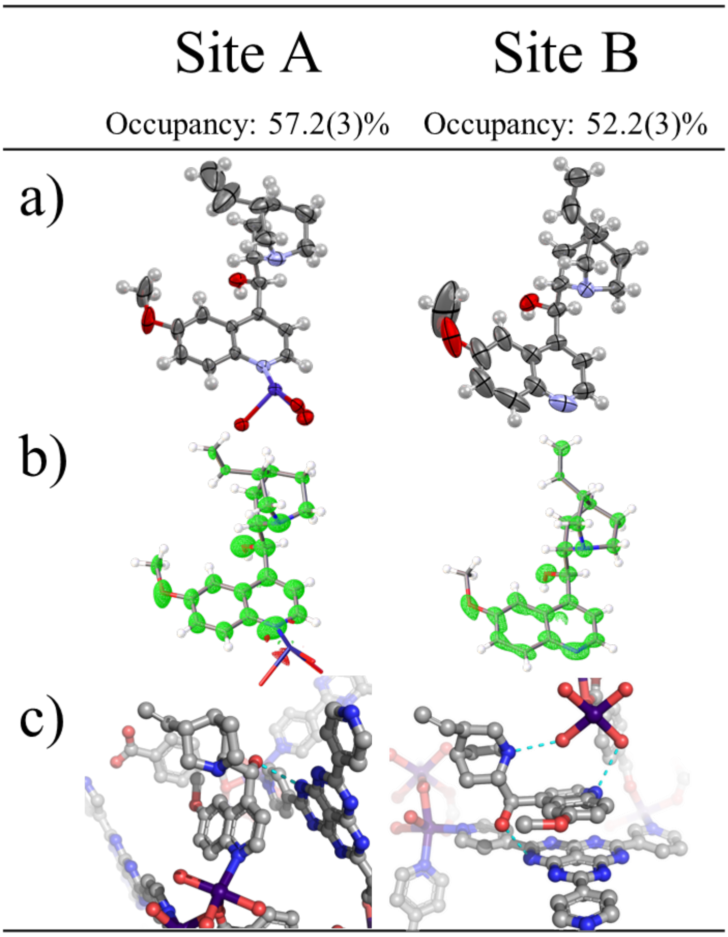

Figure S20. Quinine encapsulated structure summary. a) ORTEP diagrams with 50% probability, b) 2Fo-Fc electron density maps (the map threshold: 1.6 e/Å^3), c) the interactions between Co^{2+}, 344-TPHAP, water and quinine. Hydrogen atoms were omitted for clarity. Cyan dash lines represent hydrogen bond contacts. Atom coloring scheme: Co – purple, C – grey, H – white, O – red and N – blue.

Site A
Occupancy: 37.3(4)%

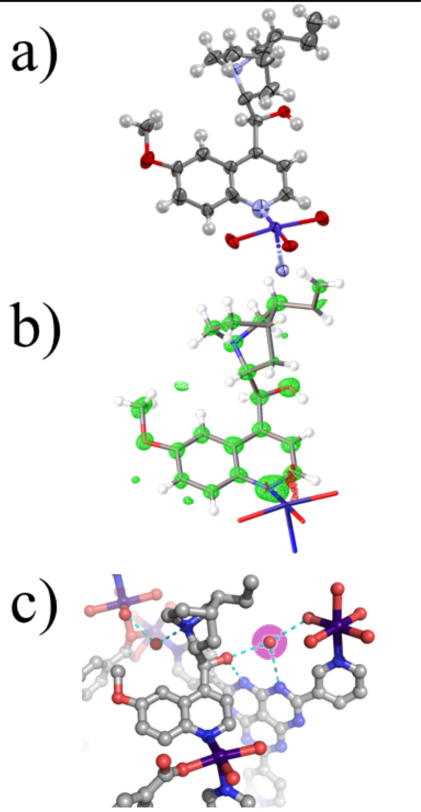

Figure S21. Quinidine encapsulated structure summary. a) ORTEP diagram with 50% probability, b) 2Fo-Fc electron density map (the map threshold: 1.7 e/Å^3), c) the interactions between Co^{2+}, 344-TPHAP, water and quinidine. Hydrogen atoms were omitted for clarity. The water site chelated between the HAP core and a water molecule coordinated to Co^{2+} was highlighted in purple. Cyan dash lines represent hydrogen bond contacts. Atom coloring scheme: Co – purple, C – grey, H – white, O – red and N – blue.

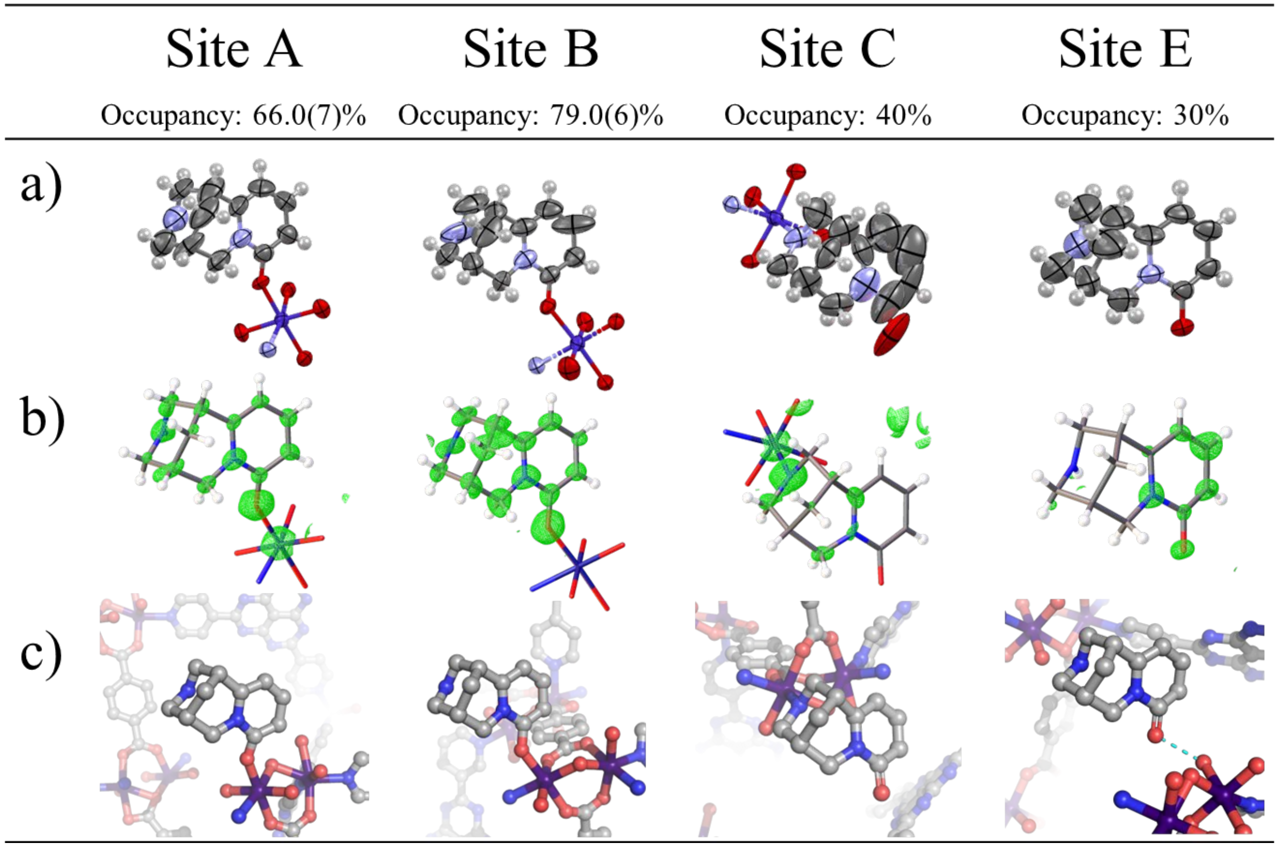

Figure S22. Cytisine encapsulated structure summary. a) ORTEP diagrams with 50% probability, b) 2Fo-Fc electron density maps (the map threshold: 1.4 e/Å^3), c) the interactions between Co^{2+}, 344-TPHAP, water and cytisine. Hydrogen atoms were omitted for clarity. Cyan dash lines represent hydrogen bond contacts. Atom coloring scheme: Co – purple, C – grey, H – white, O – red and N – blue.

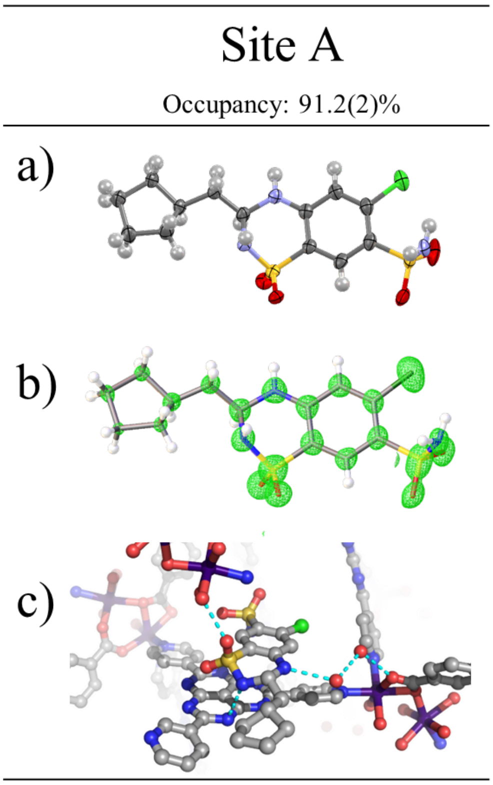

Figure S23. Cyclopenthiazide encapsulated structure summary. a) ORTEP diagram with 50% probability, b) 2Fo-Fc electron density map (the map threshold: 3 e/Å^3), c) the interactions between Co^{2+}, 344-TPHAP, water and cyclopenthiazide. Hydrogen atoms were omitted for clarity. Cyan dash lines represent hydrogen bond contacts. Atom coloring scheme: Co – purple, C – grey, H – white, O – red, N – blue, S – yellow and Cl – light green.

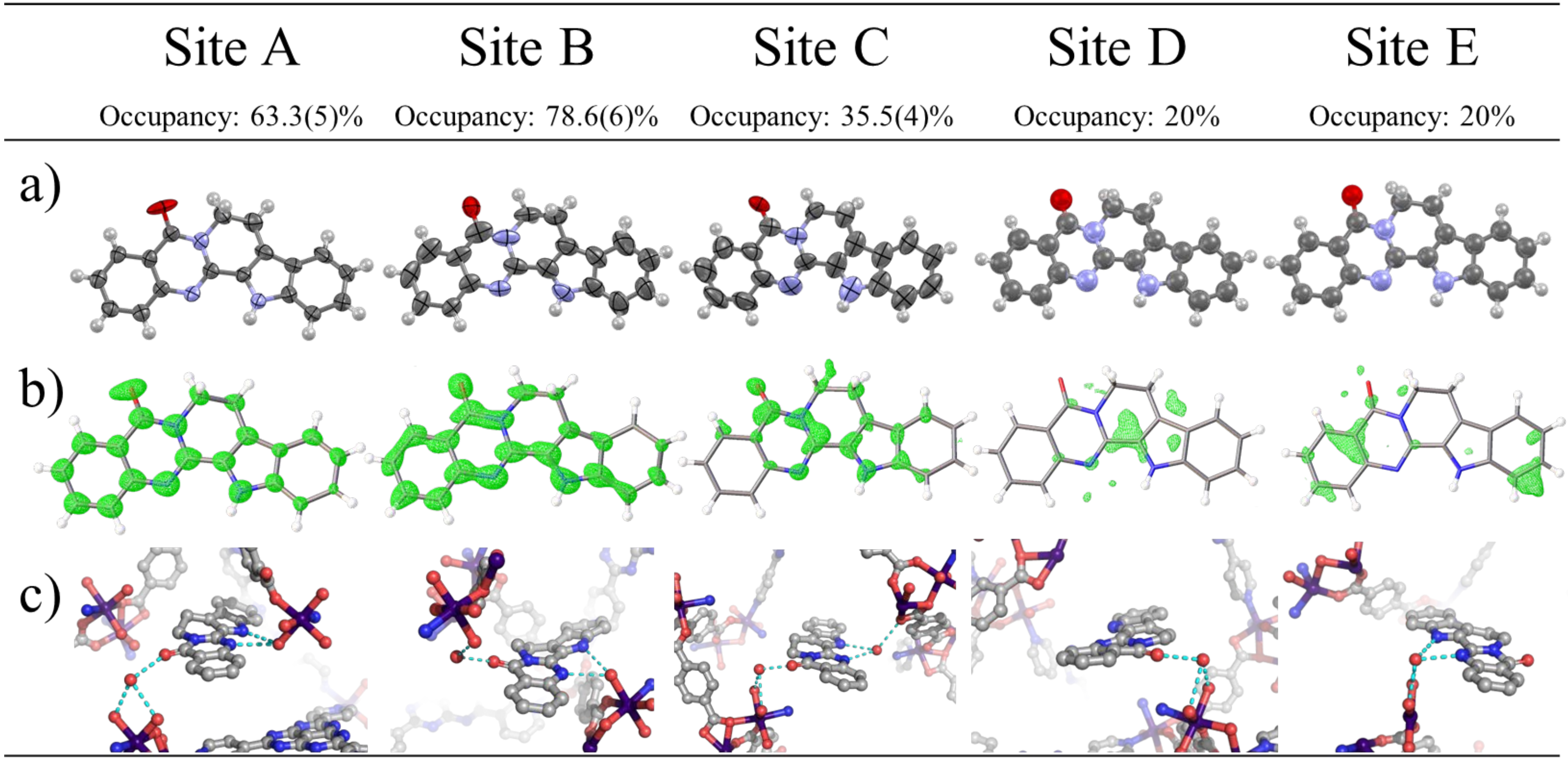

Figure S24. Rutaecarpine encapsulated structure summary. a) ORTEP diagrams with 50% probability, b) 2Fo-Fc electron density maps (the map threshold: 1.6 e/Å^3), c) The interactions between Co^{2+}, 344-TPHAP, water and rutaecarpine. Hydrogen atoms were omitted for clarity. Cyan dash lines represent hydrogen bond contacts. Atom coloring scheme: Co – purple, C – grey, H – white, O – red and N – blue.

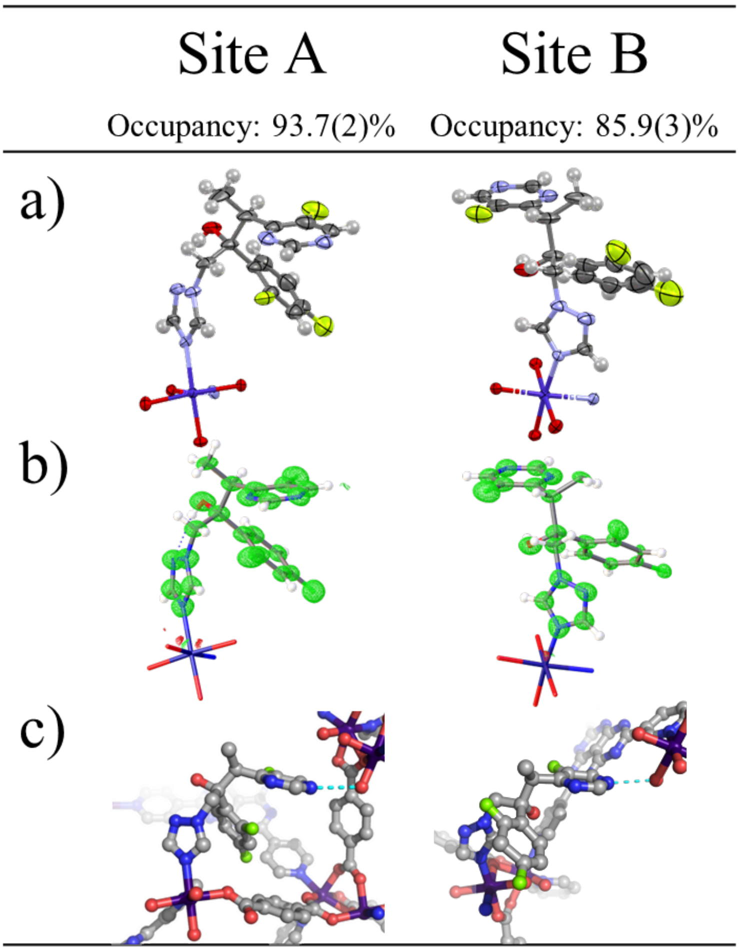

Figure S25. Voriconazole encapsulated structure summary. a) ORTEP diagrams with 50% probability, b) 2Fo-Fc electron density maps (the map threshold: 3 e/Å^3), c) the interactions between Co^{2+}, 344-TPHAP, water and voriconazole. Hydrogen atoms were omitted for clarity. Cyan dash lines represent hydrogen bond contacts. Atom coloring scheme: Co – purple, C – grey, H – white, O – red, N – blue and F – yellow-green.

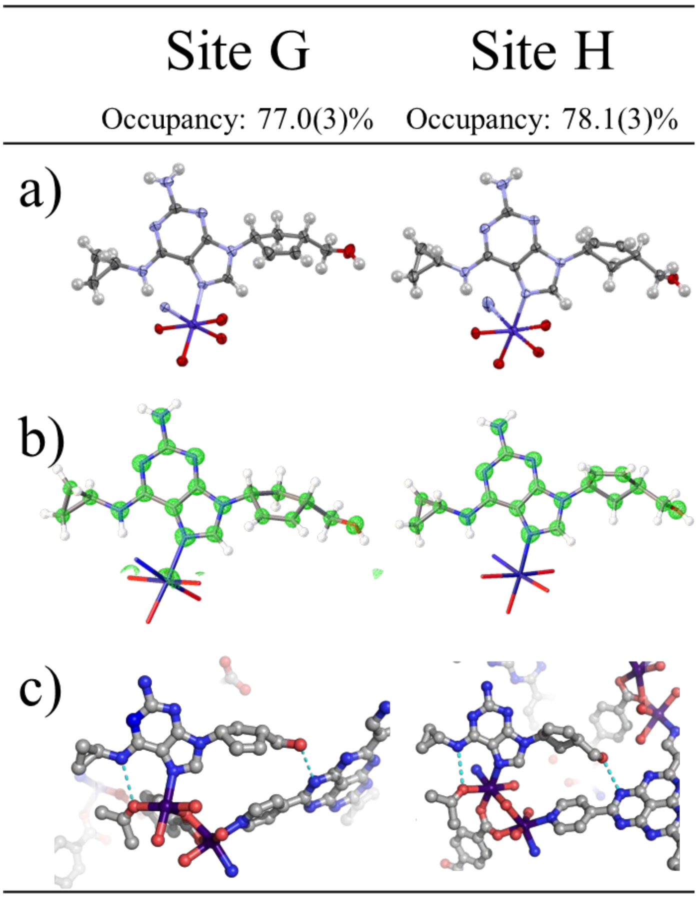

Figure S26. Abacavir encapsulated structure summary. a) ORTEP diagrams with 50% probability, b) 2Fo-Fc electron density maps (the map threshold: 3 e/Å^3), c) the interactions between Co^{2+}, 344-TPHAP, water and abacavir. Hydrogen atoms were omitted for clarity. Cyan dash lines represent hydrogen bond contacts. Atom coloring scheme: Co – purple, C – grey, H – white, O – red and N – blue.

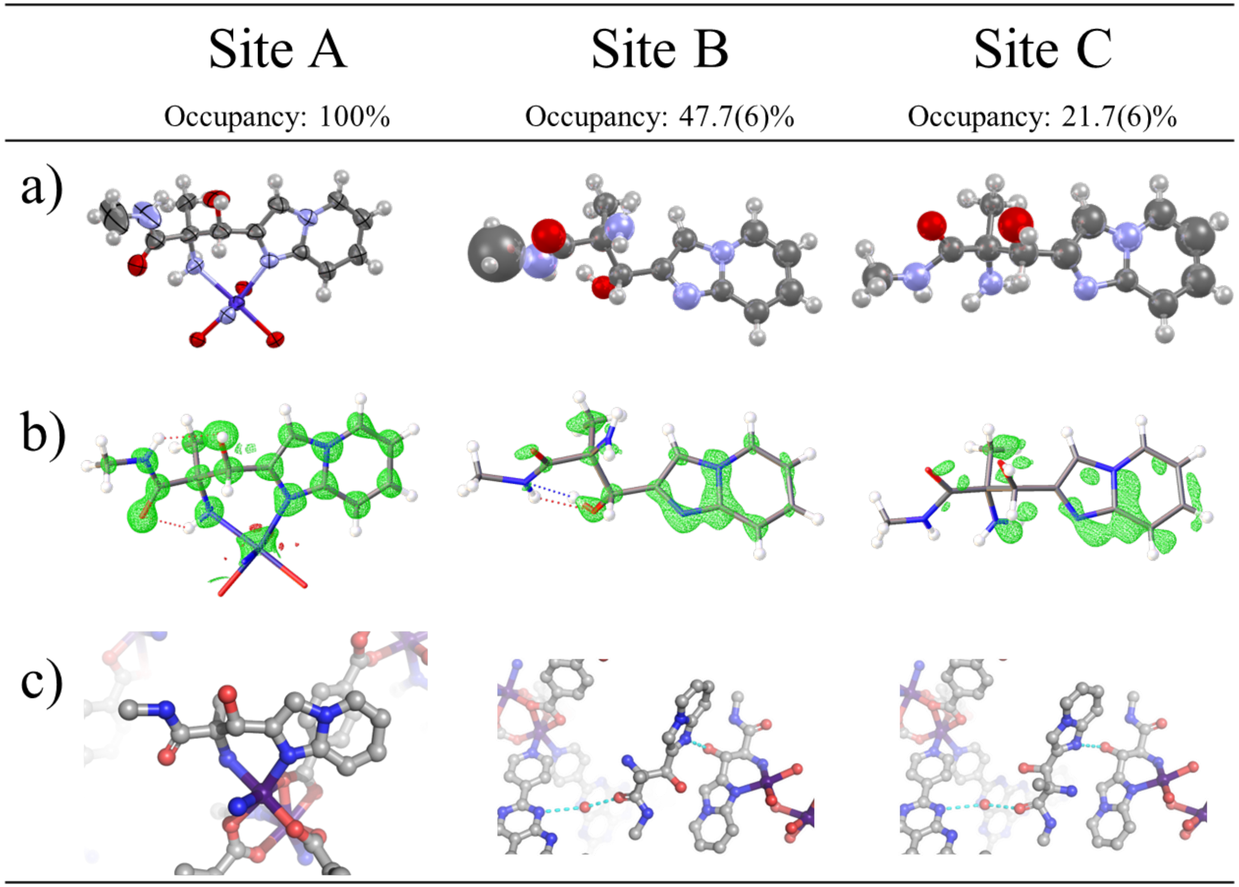

Figure S27. Compound-A encapsulated structure summary. a) ORTEP diagrams with 50% probability, b) 2Fo-Fc electron density maps (the map threshold: 2 e/Å^3), c) the interactions between Co^{2+}, 344-TPHAP, water and compound-A. Hydrogen atoms were omitted for clarity. Cyan dash lines represent hydrogen bond contacts. Atom coloring scheme: Co – purple, C – grey, H – white, O – red and N – blue.

Table S2. A list of crystallographic restraints and constraints applied during the structure analysis of encapsulated guests.

Guest name	Site	Occu. [%]	Restraints and constraints
Artemisinin	site A	46.1(4)	RIGU O1A > O20A
	site B	44.9(4)	RIGU O1B > O20B
	site C	28.0(3)	SIMU O1C > O20C
			RIGU O1C > O20C
			SAME 0.02 0.04 O1A > O20A
	site D	30.9(3)	RIGU O1D > O20D
			SAME 0.02 0.04 O1A > O20A
	site E	16.4(4)	SIMU O1F > O20F
			RIGU O1F > O20F
			SAME 0.02 0.04 O1A > O20A
Caffeine	site A	84.1(3)	None
	site B	33.8(4)	RIGU C1B > O14B
			SAME 0.02 0.04 C1A > O14A
	site C	30.6(6)	SIMU C1C > O14C
			SAME 0.02 0.04 C1A > O14A
	site D	35.5(5)	SIMU C1D > O14D
			SAME 0.02 0.04 C1A > O14A
	site E	30.7(3)	SIMU C1E > O14E
			RIGU C1E > O14E
			SAME 0.01 0.02 C1A > O14A
	site F	30.4(3)	SIMU C1G > O14G C1F > O14F
			RIGU C1F > O14F
			SAME 0.01 0.02 C1A > O14A
	site G	19.3(4)	SIMU C1G > O14G C1F > O14F
			RIGU C1G > O14G
			FLAT C1G > O14G
			SAME 0.02 0.04 C1A > O14A
Omeprazole	site A	64.7(4)	SIMU C1A > C24A
			RIGU C1A > C24A
			DFIX 1.42 C23A O22A
			DFIX 1.51 C16A C21A
	site B	37.3(6)	SIMU C1B > C24B
			RIGU C1B > C24B
			SAME 0.02 0.04 C1A > C24A
	site C	25.6(6)	SIMU C1C > C24C
			RIGU C1C > C24C
			SAME 0.02 0.04 C1A > C24A
			FLAT C15 > C22 C24
			DFIX 2.4 C23C C17C
Quinine	site A	57.2(3)	RIGU C24A C23A
	site B	52.2(3)	SIMU C9B O11B C12B
Quinidine	site A	37.3(4)	SIMU N1A > C24A
			RIGU N1A > C24A
			DFIX 1.54 C23A C18A C18A C19A
Voriconazole	site A	93.7(2)	None
	site B	85.9(3)	None
Cyclopenthiazide	site A	91.2(2)	None

Table S2(cont.). A list of crystallographic restraints and constraints applied during the structure analysis of encapsulated guests.

Guest name	Site	Occu. [%]	Restraints and constraints
Nicotine	site A	58.6(7)	SIMU N1A > C12A
			RIGU N1A > C12A
			SAME 0.02 0.04 N1B > C12B
			AFIX 66 N1A > C6A
	site B	59.1(9)	SIMU N1B > C12B
			RIGU N1B > C12B
			AFIX 66 N1B > C6B
	site C	17.8(6)	SIMU N1C > C12C
			RIGU N1C > C12C
			SAME 0.02 0.04 N1B > C12B
			AFIX 66 N1C > C6C
	site D	45.0(9)	SIMU N1D > C12D
			RIGU N1D > C12D
			SAME 0.02 0.04 N1B > C12B
			AFIX 66 N1D > C6D
	site E	61.0(7)	SIMU N1E > C12E
			RIGU N1E > C12E
			SAME 0.02 0.04 N1B > C12B
			AFIX 66 N1E > C6E
Cytisine	site A	66.0(7)	SIMU O1A > C14A
			RIGU O1A > C14A
			SAME 0.02 0.04 O1B > C14B
	site B	79.0(6)	None
	site C	40	SIMU O1C > C14C
			RIGU O1C > C14C
			SAME 0.02 0.04 O1B > C14B
	site E	30	SIMU O1E > C14E
			RIGU O1E > C14E
			SAME 0.02 0.04 O1B > C14B
Rutaecarpine	site A	63.3(5)	SIMU O1A > N22A
			RIGU O1A > N22A
	site B	78.6(6)	RIGU O1B > N22B
			SAME 0.02 0.04 O1A > N22A
	site C	35.5(4)	SIMU O1C > N22C
			RIGU O1C > N22C
			SAME 0.02 0.04 O1A > N22A
	site D	20	EADP O1D > N22D
			SAME 0.02 0.04 O1A > N22A
	site E	20	EADP O1E > N22E
			SAME 0.02 0.04 O1A > N22A
Abacavir	site G	77.0(3)	None
	site H	78.1(3)	None
Compound-A	site A	100	None
	site B	47.7(6)	SIMU C1B > C18B
			SAME 0.02 0.04 C1A > C18A
	site C	21.7(6)	SIMU C1C > C18C
			SAME 0.02 0.04 C1A > C10A
			FLAT C1C > N9C
			DFIX 1.45 O11C C10C

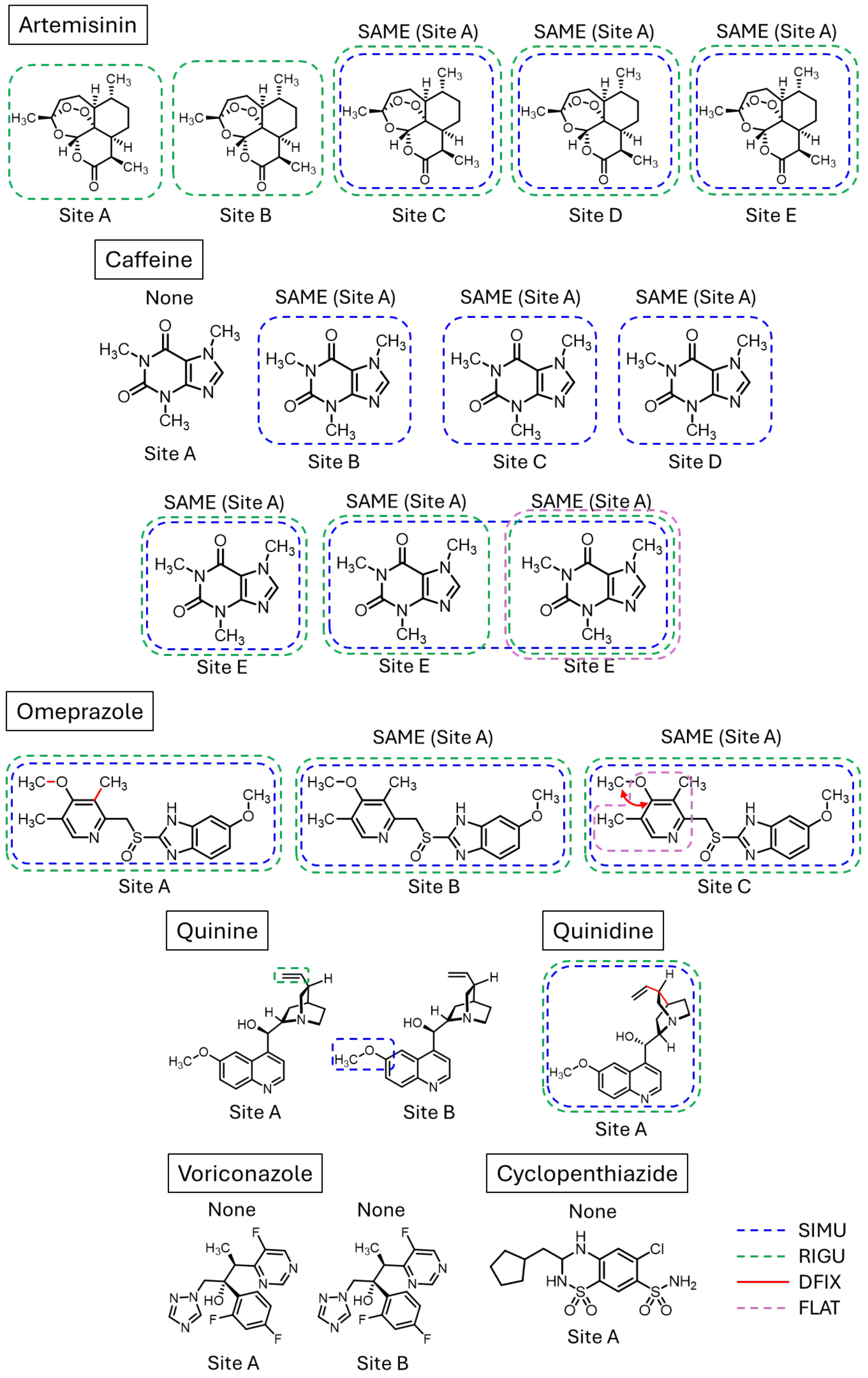

Figure S28. Visualization of crystallographic restraints and constraints.

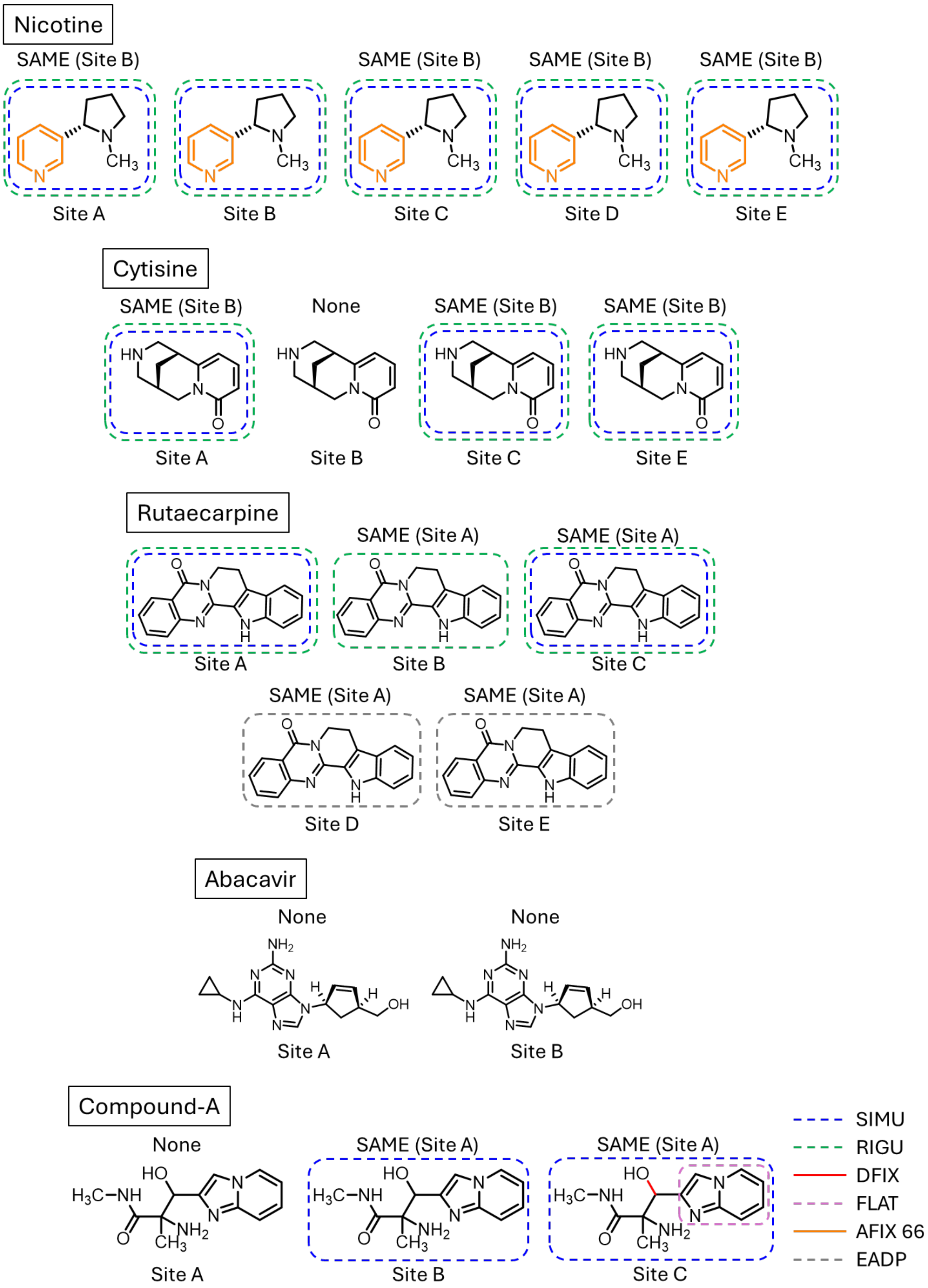

Figure S28(cont.). Visualization of crystallographic restraints and constraints.

Table S3. The lengths of Co-N coordination bonds in previously reported quinoline containing complexes obtained from the CCDC crystallographic database.

Refcode (CCDC)	Bond length (Å)	Reference
BARZIE	2.084(3)	5
BZQUCO	2.083(1)	6
BZQUCO10	2.102	7
DEPSOJ	2.054(3)	8
EQONEF	2.105(8)	9
EQONEF01	2.072(9)	9
FUFFOB	2.065(3)	10
GOLQON	2.085(2)	11
HAGPEL	2.068	12
HOGTIG	2.09(2)	13
IQUGEG	2.064(2)	14
IVOWAR	2.113(4)	15
KAKYOK01	2.053(3)	16
KEMSAW	2.044(2)	17
KEMSAW01	2.041(2)	18
LAHYAW	2.106(4)	19
MIKSAA	2.089(7)	20
MIKSEE	2.062(6)	20
NAJGEM	2.247	21
PAMSON	2.058(2)	22
PAMSUT	2.055(2)	22
PAMTAA	2.077(2)	22
PAMTEE	2.041(2)	22
PDTCOQ	2.026(2)	23
SIFZAM	2.054(7)	24
SUGQEQ	2.080(5)	25
SUGQIU	2.077(6)	25
TUSQAB	2.033(2)	26
TUSQAB01	2.064	26
TUSQAB02	2.033(3)	27
VUWZIW	2.062(3)	28
VUWZIW01	2.07	29
WOFYIB	2.014(4)	30
XEFFET	2.04(1)	31
XEFFIX	2.070(4)	31
XEFFOD	2.096(8)	31
XEFFOD01	2.087(4)	32
XEFFUJ	2.097(6)	31
XEFFUJ01	2.070(4)	20
TONXON	2.253	33

Table S4. Unit cell volumes of guest-encapsulated APF-80 structures.

Identification code	Volume / Å^3
APF-80	14260(2)
artemisinin@APF-80	14922(2)
caffeine@APF-80	14271(3)
omeprazole@APF-80	14464(9)
nicotine@APF-80	14377(5)
quinine@APF-80	14188(5)
quinidine@APF-80	14501(3)
cytisine@APF-80	14747(6)
cyclopenthiazide@APF-80	14175(5)
rutaecarpine@APF-80	14558(7)
voriconazole@APF-80	14371(1)
abacavir@APF-80	14168(2)
compound-A@APF-80	13516(4)

Table S5. Calculated binding energies and crystallographic occupancies for each guest site.

Guest name	Site	Type	Occu. [%]	Binding energy $E_{binding}$ [kJ / mol]
Artemisinin	site A	II	46.1(4)	221
	site B	II	44.9(4)	207
	site C	II	28.0(3)	164
	site D	II	30.9(3)	136
	site E	III	16.4(4)	141
Caffeine	site A	II	84.1(3)	227
	site B	II	33.8(4)	215
	site C	II	30.6(6)	230
	site D	II	35.5(5)	235
	site E	II	30.7(3)	107
	site F	II	30.4(4)	145
	site G	I	19.3(5)	80.0
Omeprazole	site A	V	64.7(4)	352
	site B	V	37.3(6)	392
	site C	V	25.6(6)	322
Nicotine	site A	III	58.6(7)	207
	site B	III	59.1(9)	200
	site C	I	17.8(6)	108
	site D	III	45.0(9)	186
	site E	III	61.0(7)	216
Quinine	site A	III	57.2(3)	217
	site B	II	52.2(3)	224
Quinidine	site A	II	37.3(4)	248
Cytisine	site A	III	66.0(7)	212
	site B	III	79.0(6)	209
	site C	III	40	164
	site E	II	30	141
Cyclopenthiazide	site A	II	91.2(2)	302
Rutaecarpine	site A	II	63.3(5)	177
	site B	II	78.6(6)	201
	site C	II	35.5(4)	188
	site D	II	20	66.0
	site E	II	20	176
Voriconazole	site A	III	93.7(2)	251
	site B	III	85.9(3)	255
Abacavir	site G	IV	77.0(3)	292
	site H	IV	78.1(3)	258
Compound-A	site A	V	100	339
	site B	II	47.7(6)	189
	site C	II	21.7(6)	157

Table S6. Crystallographic tables.

Identification code	3PP	APF-80
Empirical formula	$C_{20}H_{16}N_{12}$	$C_{51.58}H_{57.56}Co_2N_{13.4}O_{13.33}$
Formula weight	424.45	1196.31
Temperature / K	123	95
Crystal system	triclinic	orthorhombic
Space group	*P-1*	*Pbca*
a / Å	7.0900(7)	26.286(1)
b / Å	12.307(1)	16.708(1)
c / Å	12.962(1)	32.468(3)
α / °	112.515(8)	90
β / °	104.756(7)	90
γ / °	100.322(8)	90
Volume / $Å^3$	960.60(2)	14260(2)
Z	2	8
ρ_{calc}g / cm^3	1.467	1.114
μ / mm^{-1}	0.099	0.603
F(000)	440.0	4972.0
Crystal size / mm^3	0.223 × 0.05 × 0.037	0.2 × 0.2 × 0.1
Radiation	Mo Kα (λ = 0.71073)	synchrotron (λ = 0.75)
2Θ range for data collection / °	3.64 to 48.87	3.112 to 71.048
Index ranges	-8 ≤ h ≤ 8, -14 ≤ k ≤ 14, -15 ≤ l ≤ 15	-40 ≤ h ≤ 37, -25 ≤ k ≤ 25, -49 ≤ l ≤ 50
Reflections collected	9518	151414
Independent reflections	3167 [R_{int} = 0.0823, R_{sigma} = 0.1104]	27057 [R_{int} = 0.0126, R_{sigma} = 0.0082]
Data / restraints / parameters	3167/0/289	27057/2346/1148
Goodness-of-fit on F^2	1.022	1.036
Final R indexes [I >= 2σ (I)]	R_1 = 0.1002, wR_2 = 0.2682	R_1 = 0.0529, wR_2 = 0.1672
Final R indexes [all data]	R_1 = 0.1608, wR_2 = 0.3240	R_1 = 0.0535, wR_2 = 0.1679
Largest diff. peak / hole / e $Å^{-3}$	0.67/-0.55	0.81/-0.72
Flack parameter	-	-
CCDC	2446306	2446307

Table S6(cont.). Crystallographic tables.

Identification code	artemisinin@APF-80	caffeine@APF-80
Empirical formula	$C_{93.92}H_{74.39}Co_4N_{18}O_{26.61}$	$C_{55.75}H_{45.64}Co_2N_{19.57}O_{17.52}$
Formula weight	2116.72	1387.92
Temperature / K	95	95
Crystal system	orthorhombic	orthorhombic
Space group	$P2_12_12_1$	*Pbca*
a / Å	17.538(3)	26.279(1)
b / Å	25.719(1)	16.588(4)
c / Å	33.083(1)	32.739(2)
α / °	90	90
β / °	90	90
γ / °	90	90
Volume / Å^3	14922(2)	14271(3)
Z	4	8
ρ_{calc}g / cm^3	0.942	1.292
μ / mm^{-1}	0.567	0.621
F(000)	4339.0	5690.0
Crystal size / mm^3	0.15 × 0.15 × 0.1	0.25 × 0.05 × 0.05
Radiation	synchrotron (λ = 0.75)	synchrotron (λ = 0.75)
2θ range for data collection / °	2.116 to 71.158	3.094 to 71.094
Index ranges	$-21 \leq h \leq 26$, $-39 \leq k \leq 39$, $-47 \leq l \leq 47$	$-39 \leq h \leq 38$, $-25 \leq k \leq 25$, $-48 \leq l \leq 48$
Reflections collected	161036	300969
Independent reflections	53573 [R_{int} = 0.0188, R_{sigma} = 0.0202]	26613 [R_{int} = 0.0297, R_{sigma} = 0.0135]
Data / restraints / parameters	53573/1557/1895	26613/1262/1397
Goodness-of-fit on F^2	1.056	1.071
Final R indexes [I >= 2σ (I)]	R_1 = 0.0751, wR_2 = 0.2235	R_1 = 0.0582, wR_2 = 0.1811
Final R indexes [all data]	R_1 = 0.0824, wR_2 = 0.2378	R_1 = 0.0608, wR_2 = 0.1844
Largest diff. peak / hole / e Å^{-3}	0.97/-1.44	0.78/-0.82
Flack parameter	0.034(3)	-
CCDC	2446376	2446377

Table S6(cont.). Crystallographic tables.

Identification code	omeprazole@APF-80	nicotine@APF-80
Empirical formula	$C_{95.57}H_{72}Co_4N_{21.83}O_{20.63}S_{1.28}$	$C_{94.95}H_{76.21}Co_4N_{22.83}O_{14.17}$
Formula weight	2132.99	1999.49
Temperature / K	95	95
Crystal system	orthorhombic	orthorhombic
Space group	$Pca2_1$	$P2_12_12_1$
a / Å	26.315(5)	26.381(5)
b / Å	32.704(7)	16.866(4)
c / Å	16.807(9)	32.312(5)
α / °	90	90
β / °	90	90
γ / °	90	90
Volume / Å^3	14464(9)	14377(5)
Z	4	4
ρ_{calc}g / cm^3	0.979	0.924
μ / mm^{-1}	0.603	0.579
F(000)	4367.0	4108.0
Crystal size / mm^3	0.075 × 0.075 × 0.075	0.2 × 0.2 × 0.05
Radiation	synchrotron (λ = 0.75)	synchrotron (λ = 0.75)
2Θ range for data collection / °	2.096 to 71.086	2.66 to 71.104
Index ranges	$-39 \le h \le 39$, $-48 \le k \le 48$, $-22 \le l \le 22$	$-40 \le h \le 37$, $-25 \le k \le 26$, $-50 \le l \le 49$
Reflections collected	309564	155618
Independent reflections	51932 [R_{int} = 0.0257, R_{sigma} = 0.0178]	53107 [R_{int} = 0.0483, R_{sigma} = 0.0498]
Data / restraints / parameters	51932/1462/1791	53107/1176/1511
Goodness-of-fit on F^2	1.177	1.039
Final R indexes [I >= 2σ (I)]	R_1 = 0.0940, wR_2 = 0.2745	R_1 = 0.0896, wR_2 = 0.2613
Final R indexes [all data]	R_1 = 0.1150, wR_2 = 0.3023	R_1 = 0.1365, wR_2 = 0.3140
Largest diff. peak / hole / e Å^{-3}	0.74/-1.73	0.90/-1.00
Flack parameter	0.501(2)	0.291(9)
CCDC	2446378	2446379

Table S6(cont.). Crystallographic tables.

Identification code	quinine@APF-80	quinidine@APF-80
Empirical formula	$C_{98.43}H_{80.75}Co_4N_{20.19}O_{21.41}$	$C_{82.96}H_{60.95}Co_4N_{18.75}O_{22.15}$
Formula weight	2124.60	1910.56
Temperature / K	95	95
Crystal system	orthorhombic	orthorhombic
Space group	$P2_12_12_1$	$P2_12_12_1$
a / Å	17.080(6)	16.988(3)
b / Å	25.816(9)	26.198(7)
c / Å	32.176(8)	32.583(2)
α / °	90	90
β / °	90	90
γ / °	90	90
Volume / Å^3	14188(5)	14501(3)
Z	4	4
ρ_{calc}g / cm^3	0.995	0.875
μ / mm^{-1}	0.594	0.575
F(000)	4368.0	3901.0
Crystal size / mm^3	0.2 × 0.2 × 0.15	0.25 × 0.2 × 0.1
Radiation	synchrotron (λ = 0.75)	synchrotron (λ = 0.75)
2Θ range for data collection / °	2.134 to 71.052	2.104 to 71.082
Index ranges	$-23 \le h \le 23, -39 \le k \le 39, -48 \le l \le 48$	$-25 \le h \le 25, -32 \le k \le 32, -48 \le l \le 48$
Reflections collected	310880	320547
Independent reflections	52779 [R_{int} = 0.0116, R_{sigma} = 0.0078]	49584 [R_{int} = 0.0129, R_{sigma} = 0.0078]
Data / restraints / parameters	52779/211/1660	49584/599/1490
Goodness-of-fit on F^2	1.033	1.046
Final R indexes [I >= 2σ (I)]	R_1 = 0.0668, wR_2 = 0.2003	R_1 = 0.0679, wR_2 = 0.2193
Final R indexes [all data]	R_1 = 0.0690, wR_2 = 0.2043	R_1 = 0.0694, wR_2 = 0.2214
Largest diff. peak / hole / e Å^{-3}	0.86/-1.71	0.87/-1.11
Flack parameter	0.044(2)	0.128(3)
CCDC	2446380	2446381

Table S6(cont.). Crystallographic tables.

Identification code	cytisine@APF-80	cyclopenthiazide@APF-80
Empirical formula	$C_{96.25}H_{75.69}Co_4N_{22.3}O_{17.05}$	$C_{55.93}H_{58.27}Cl_{0.89}Co_2N_{11.72}O_{18.24}S_{1.8}$
Formula weight	2053.14	1393.70
Temperature / K	95	95
Crystal system	orthorhombic	orthorhombic
Space group	$P2_12_12_1$	*Pbca*
a / Å	17.402(7)	25.850(2)
b / Å	25.829(2)	16.856(6)
c / Å	32.810(2)	32.531(2)
α / °	90	90
β / °	90	90
γ / °	90	90
Volume / $Å^3$	14747(6)	14175(5)
Z	4	8
$\rho_{calc}g / cm^3$	0.925	1.306
μ / mm^{-1}	0.567	0.720
F(000)	4215.0	5758.0
Crystal size / mm^3	0.075 × 0.075 × 0.075	0.075 × 0.075 × 0.075
Radiation	synchrotron (λ = 0.75)	synchrotron (λ = 0.75)
2Θ range for data collection / °	2.118 to 71.048	2.642 to 71.076
Index ranges	$-23 \leq h \leq 23$, $-38 \leq k \leq 38$, $-50 \leq l \leq 50$	$-38 \leq h \leq 38$, $-21 \leq k \leq 21$, $-48 \leq l \leq 48$
Reflections collected	323913	304434
Independent reflections	53435 [R_{int} = 0.0243, R_{sigma} = 0.0161]	24725 [R_{int} = 0.0187, R_{sigma} = 0.0079]
Data / restraints / parameters	53435/1023/1593	24725/428/1254
Goodness-of-fit on F^2	1.101	1.047
Final R indexes [I >= 2σ (I)]	R_1 = 0.0848, wR_2 = 0.2513	R_1 = 0.0388, wR_2 = 0.1156
Final R indexes [all data]	R_1 = 0.1016, wR_2 = 0.2743	R_1 = 0.0408, wR_2 = 0.1173
Largest diff. peak / hole / e $Å^{-3}$	0.76/-0.96	0.70/-0.79
Flack parameter	0.063(4)	-
CCDC	2446382	2446383

Table S6(cont.). Crystallographic tables.

Identification code	rutaecarpine@APF-80	voriconazole@APF-80
Empirical formula	$C_{112.28}H_{76.05}Co_4N_{24.52}O_{23.34}$	$C_{116.93}H_{104.53}Co_4F_{5.39}N_{26.98}O_{20.93}$
Formula weight	2377.75	2560.63
Temperature / K	95	95
Crystal system	orthorhombic	orthorhombic
Space group	*Pca2₁* → $Pca2_1$	$P2_12_12_1$
a / Å	32.581(2)	17.014(1)
b / Å	17.072(9)	26.148(9)
c / Å	26.173(3)	32.303(7)
α / °	90	90
β / °	90	90
γ / °	90	90
Volume / Å^3	14558(7)	14371(1)
Z	4	4
ρ_{calc}g / cm^3	1.085	1.184
μ / mm^{-1}	0.588	0.606
F(000)	4864.0	5276.0
Crystal size / mm^3	0.2 × 0.2 × 0.075	0.075 × 0.075 × 0.075
Radiation	synchrotron (λ = 0.75)	synchrotron (λ = 0.75)
2Θ range for data collection / °	2.638 to 71.088	2.66 to 71.112
Index ranges	-48 ≤ h ≤ 48, -20 ≤ k ≤ 20, -39 ≤ l ≤ 39	-20 ≤ h ≤ 20, -38 ≤ k ≤ 38, -48 ≤ l ≤ 48
Reflections collected	306927	318140
Independent reflections	48256 [R_{int} = 0.0256, R_{sigma} = 0.0159]	48884 [R_{int} = 0.0171, R_{sigma} = 0.0101]
Data / restraints / parameters	48256/1631/1833	48884/277/1829
Goodness-of-fit on F^2	1.028	1.028
Final R indexes [I >= 2σ (I)]	R_1 = 0.0849, wR_2 = 0.2382	R_1 = 0.0471, wR_2 = 0.1422
Final R indexes [all data]	R_1 = 0.0944, wR_2 = 0.2548	R_1 = 0.0481, wR_2 = 0.1434
Largest diff. peak / hole / e Å^{-3}	0.90/-2.19	0.81/-0.80
Flack parameter	0.499(2)	0.017(2)
CCDC	2446384	2446385

Table S6(cont.). Crystallographic tables.

Identification code	abacavir@APF-80	compound-A@APF-80
Empirical formula	$C_{106.86}H_{98.22}Co_4N_{27.3}O_{24.88}$	$C_{55.24}H_{46.92}Co_2N_{15.78}O_{12.95}$
Formula weight	2398.65	1256.87
Temperature / K	95	95
Crystal system	orthorhombic	orthorhombic
Space group	$P2_12_12_1$	*Pbca*
a / Å	16.554(2)	24.607(1)
b / Å	26.342(1)	17.298(5)
c / Å	32.490(3)	31.753(1)
α / °	90	90
β / °	90	90
γ / °	90	90
Volume / $Å^3$	14168(2)	13516(4)
Z	4	8
$\rho_{calc}g / cm^3$	1.125	1.235
μ / mm^{-1}	0.606	0.641
F(000)	4950.0	5171.0
Crystal size / mm^3	0.4 × 0.3 × 0.2	0.3 × 0.2 × 0.2
Radiation	synchrotron (λ = 0.75)	synchrotron (λ = 0.75)
2Θ range for data collection / °	2.1 to 71.09	2.706 to 71.088
Index ranges	-25 ≤ h ≤ 25, -38 ≤ k ≤ 38, -48 ≤ l ≤ 48	-36 ≤ h ≤ 36, -20 ≤ k ≤ 20, -47 ≤ l ≤ 47
Reflections collected	308244	284529
Independent reflections	53266 [R_{int} = 0.0184, R_{sigma} = 0.0122]	22950 [R_{int} = 0.0455, R_{sigma} = 0.0190]
Data / restraints / parameters	53266/418/1960	22950/154/859
Goodness-of-fit on F^2	1.048	1.022
Final R indexes [I >= 2σ (I)]	R_1 = 0.0600, wR_2 = 0.1882	R_1 = 0.0783, wR_2 = 0.2377
Final R indexes [all data]	R_1 = 0.0622, wR_2 = 0.1903	R_1 = 0.1017, wR_2 = 0.2673
Largest diff. peak / hole / e $Å^{-3}$	0.84/-1.46	0.92/-0.94
Flack parameter	0.092(2)	-
CCDC	2446386	2446308

A- or B-level checkCIF alerts and their responses.

CCDC 2446306 (3PP)

No alerts were reported.

CCDC 2446307 (APF-80)

_vrf_PLAT220_APF-80

;

PROBLEM: NonSolvent Resd 1 C Ueq(max)/Ueq(min) Range 6.1 Ratio

RESPONSE:

This data includes guest molecules encapsulated in MOF.

Compared to framework, the position of the encapsulated guest in the pore is not fixed in the crystal. Therefore, the thermal ellipsoid is inevitably larger than in the framework, because it is affected by the residual electron density of the solvent and thermal vibration.

;

CCDC 2446376 (artemisinin@APF-80)

_vrf_PLAT602_artemisinin@APF-80

;

PROBLEM: Solvent Accessible VOID(S) in the Unit Cell .... ! Check

RESPONSE:

We assigned solvents inside the pore as many as possible. However, several solvents could not be modeled due to severe disorder.

;

_vrf_PLAT049_artemisinin@APF-80

;

PROBLEM: Calculated Density Less Than 1.0 gcm-3 .... 0.9423 Check

RESPONSE:

In the pores of the MOF, there are areas where solvent molecules are severely disordered, resulting in an apparent loss of mass.

;

_vrf_PLAT220_artemisinin@APF-80

;

PROBLEM: NonSolvent Resd 1 C Ueq(max)/Ueq(min) Range 7.0 Ratio

RESPONSE:

This data includes guest molecules encapsulated in MOF. Compared to framework, the position of the encapsulated guest in the pore is not fixed in the crystal. Therefore, the thermal ellipsoid is inevitably larger than in the framework, because it is affected by the

residual electron density of the solvent and thermal vibration.

;

_vrf_PLAT430_artemisinin@APF-80

;

PROBLEM: Short Inter D...A Contact O1W ..O6W . 2.58 Ang.

RESPONSE:

These oxygen atoms are components of the water in the pore of the framework. Since it was difficult to determine the static position of the hydrogen atoms of the water without clear interactions, the model has no hydrogen.

;

CCDC 2446377 (caffeine@APF-80)

_vrf_PLAT430_caffeine@APF-80

;

PROBLEM: Short Inter D...A Contact O3W ..O11W . 2.78 Ang.

RESPONSE:

These oxygen atoms are components of the water in the pore of the framework. Since it was difficult to determine the static position of the hydrogen atoms of the water without clear interactions, the model has no hydrogen.

;

CCDC 2446378 (omeprazole@APF-80)

_vrf_PLAT602_omeprazole@APF-80

;

PROBLEM: Solvent Accessible VOID(S) in the Unit Cell .... ! Check

RESPONSE:

We assigned solvents inside the pore as much as possible. However, several solvents could not be modeled due to severe disorder.

;

_vrf_PLAT049_omeprazole@APF-80

;

PROBLEM: Calculated Density Less Than 1.0 gcm-3 .... 0.9652 Check

RESPONSE:

In the pores of the MOF, there are areas where solvent molecules are severely disordered, resulting in an apparent loss of mass.

;

_vrf_PLAT220_omeprazole@APF-80

;

PROBLEM: NonSolvent Resd 1 C Ueq(max)/Ueq(min) Range 7.7 Ratio

RESPONSE:

This data includes guest molecules encapsulated in MOF. Compared to framework, the position of the encapsulated guest in the pore is not fixed in the crystal. Therefore, the thermal ellipsoid is inevitably larger than in the framework, because it is affected by the residual electron density of the solvent and thermal vibration.

;

_vrf_PLAT430_omeprazole@APF-80

;

PROBLEM: Short Inter D...A Contact O7W ..N48 . 2.68 Ang.

RESPONSE:

These oxygen atoms are components of the water in the pore of the framework. Since it was difficult to determine the static position of the hydrogen atoms of the water without clear interactions, the model has no hydrogen.

;

CCDC 2446379 (nicotine@APF-80)

_vrf_PLAT602_nicotine@APF-80

;

PROBLEM: Solvent Accessible VOID(S) in the Unit Cell .... ! Check

RESPONSE:

We assigned solvents inside the pore as much as possible. However, several solvents could not be modeled due to severe disorder.

;

_vrf_PLAT049_nicotine@APF-80

;

PROBLEM: Calculated Density Less Than 1.0 gcm-3 .... 0.9238 Check

RESPONSE:

In the pores of the MOF, there are areas where solvent molecules are severely disordered, resulting in an apparent loss of mass.

;

_vrf_PLAT220_nicotine@APF-80

;

PROBLEM: NonSolvent Resd 1 C Ueq(max)/Ueq(min) Range 9.4 Ratio

RESPONSE:

This data includes guest molecules encapsulated in MOF. Compared to framework, the position of the encapsulated guest in the pore is not fixed in the crystal. Therefore, the

thermal ellipsoid is inevitably larger than in the framework, because it is affected by the residual electron density of the solvent and thermal vibration.

;

CCDC 2446380 (quinine@APF-80)

_vrf_PLAT049_quinine@APF-80

;

PROBLEM: Calculated Density Less Than 1.0 gcm-3 .... 0.9945 Check

RESPONSE:

In the pores of the MOF, there are areas where solvent molecules are severely disordered, resulting in an apparent loss of mass.

;

_vrf_PLAT220_quinine@APF-80

;

PROBLEM: NonSolvent Resd 1 C Ueq(max)/Ueq(min) Range 6.9 Ratio

RESPONSE:

This data includes guest molecules encapsulated in MOF. Compared to framework, the position of the encapsulated guest in the pore is not fixed in the crystal. Therefore, the thermal ellipsoid is inevitably larger than in the framework, because it is affected by the residual electron density of the solvent and
thermal vibration.

;

_vrf_PLAT430_quinine@APF-80

;

PROBLEM: Short Inter D...A Contact O1W ..N16B . 2.83 Ang.

RESPONSE:

These oxygen atoms are components of the water in the pore of the framework. Since it was difficult to determine the static position of the hydrogen atoms of the water without clear interactions, the model has no hydrogen.

;

_vrf_PLAT601_quinine@APF-80

;

PROBLEM: Unit Cell Contains Solvent Accessible VOIDS <= 117 Ang**3

RESPONSE:

We assigned solvents inside the pore as much as possible. However, several solvents could not be modeled due to severe disorder.

;

CCDC 2446381 (quinidine@APF-80)

_vrf_PLAT602_quinidine@APF-80

;

PROBLEM: Solvent Accessible VOID(S) in the Unit Cell .... ! Check

RESPONSE:

We assigned solvents inside the pore as much as possible. However, several solvents could not be modeled due to severe disorder.

;

_vrf_PLAT049_quinidine@APF-80

;

PROBLEM: Calculated Density Less Than 1.0 gcm-3 .... 0.8751 Check

RESPONSE:

In the pores of the MOF, there are areas where solvent molecules are severely disordered, resulting in an apparent loss of mass.

;

_vrf_PLAT430_quinidine@APF-80

;

PROBLEM: Short Inter D...A Contact O1W ..O6W . 2.77 Ang.

RESPONSE:

These oxygen atoms are components of the water in the pore of the framework. Since it was difficult to determine the static position of the hydrogen atoms of the water without clear interactions, the model has no hydrogen.

;

CCDC 2446382 (cytisine@APF-80)

_vrf_PLAT602_cytisine@APF-80

;

PROBLEM: Solvent Accessible VOID(S) in the Unit Cell .... ! Check

RESPONSE:

We assigned solvents inside the pore as much as possible. However, several solvents could not be modeled due to severe disorder.

;

_vrf_PLAT049_cytisine@APF-80

;

PROBLEM: Calculated Density Less Than 1.0 gcm-3 .... 0.9248 Check

RESPONSE:

In the pores of the MOF, there are areas where solvent molecules are severely disordered,

resulting in an apparent loss of mass.

;

_vrf_PLAT220_cytisine@APF-80

;

PROBLEM: NonSolvent Resd 1 C Ueq(max)/Ueq(min) Range 6.1 Ratio

RESPONSE:

This data includes guest molecules encapsulated in MOF. Compared to framework, the position of the encapsulated guest in the pore is not fixed in the crystal. Therefore, the thermal ellipsoid is inevitably larger than in the framework because it is affected by the residual electron density of the solvent and thermal vibration.

;

CCDC 2446383 (cyclopenthiazide@APF-80)

_vrf_PLAT430_cyclopenthiazide@APF-80

;

PROBLEM: Short Inter D...A Contact O1W ..O8W . 2.62 Ang.

RESPONSE:

These oxygen atoms are components of the water in the pore of the framework. Since it was difficult to determine the static position of the hydrogen atoms of the water without clear interactions, the model has no hydrogen.

;

CCDC 2446384 (rutecarpine@APF-80)

_vrf_PLAT029_rutecarpine@APF-80

;

PROBLEM: _diffrn_measured_fraction_theta_full value Low . 0.951 Why?

RESPONSE:

In this measurement, the crystal was measured by omega scan.

Omega scan measurement provided the best data quality, and it is suitable for this research.

However, diffraction data set of this crystal was slightly not enough to cover the full sphere.

;

_vrf_PLAT430_rutecarpine@APF-80

;

PROBLEM: Short Inter D...A Contact O7W ..N42 . 2.81 Ang.

RESPONSE:

These oxygen atoms are components of the water in the pore of the framework. Since it was difficult to determine the static position of the hydrogen atoms of the water without clear interactions, the model has no hydrogen.

;

_vrf_PLAT601_rutecarpine@APF-80

;

PROBLEM: Unit Cell Contains Solvent Accessible VOIDS <= 139 Ang**3

RESPONSE:

We assigned solvents inside the pore as much as possible. However, several solvents could not be modeled due to severe disorder.

;

CCDC 2446385 (voriconazole@APF-80)

_vrf_PLAT430_voriconazole@APF-80

;

PROBLEM: Short Inter D...A Contact O1H ..O2W . 2.74 Ang.

RESPONSE:

These oxygen atoms are components of the water in the pore of the framework. Since it was difficult to determine the static position of the hydrogen atoms of the water without clear interactions, the model has no hydrogen.

;

CCDC 2446386 (abacavir@APF-80)

_vrf_PLAT220_abacavir@APF-80

;

PROBLEM: NonSolvent Resd 1 O Ueq(max)/Ueq(min) Range 7.0 Ratio

RESPONSE:

This data includes guest molecules encapsulated in MOF. Compared to framework, the position of the encapsulated guest in the pore is not fixed in the crystal. Therefore, the thermal ellipsoid is inevitably larger than in the framework because it is affected by the residual electron density of the solvent and thermal vibration.

;

CCDC 2446308 (compound-A@APF-80)

_vrf_PLAT220_compound-A@APF-80

;

PROBLEM: NonSolvent Resd 1 C Ueq(max)/Ueq(min) Range 6.6 Ratio

RESPONSE:

This data includes guest molecules encapsulated in MOF. Compared to framework, the position of the encapsulated guest in the pore is not fixed in the crystal. Therefore, the thermal ellipsoid is inevitably larger than in the framework because it is affected by the residual electron density of the solvent and thermal vibration.

;

References

(1) Kabsch, W. *XDS*. *Acta Crystallogr. Sect. D* **2010**, *66* (2), 125–132.

(2) Sheldrick, G. M. *SHELXT* – Integrated Space-Group and Crystal-Structure Determination. *Acta Crystallogr. Sect. Found. Adv.* **2015**, *71* (1), 3–8.

(3) Sheldrick, G. M. Crystal Structure Refinement with *SHELXL*. *Acta Crystallogr. Sect. C Struct. Chem.* **2015**, *71* (1), 3–8.

(4) Dolomanov, O. V.; Bourhis, L. J.; Gildea, R. J.; Howard, J. a. K.; Puschmann, H. OLEX2: A Complete Structure Solution, Refinement and Analysis Program. *J. Appl. Crystallogr.* **2009**, *42* (2), 339–341.

(5) Moulton, B.; Abourahma, H.; Bradner, M. W.; Lu, J.; McManus, G. J.; Zaworotko, M. J. A New $6^5.8$ Topology and a Distorted $6^5.8$ $CdSO_4$ Topology: Two New Supramolecular Isomers of $[M_2(Bdc)_2(L)_2]_n$ Coordination polymers. *Chem. Commun.* **2003**, No. 12, 1342.

(6) Hudák, J.; Boča, R.; Dlháň, Ľ.; Kožíšek, J.; Moncoľ, J. Structure and Magnetism of Mono-, Di-, and Trinuclear Benzoato Cobalt(II) Complexes. *Polyhedron* **2011**, *30* (7), 1367–1373.

(7) Catterick, J.; Hursthouse, M. B.; Thornton, P.; Welch, A. J. Crystal and Molecular Structure of Tetra-μ-Benzoato-Bisquinolinedi-Cobalt(II), a Binuclear Cobalt(II) Carboxylate. *J Chem Soc Dalton Trans* **1977**, No. 3, 223–226.

(8) Brescia, T. K.; Mulosmani, K.; Gulati, S.; Athanasopoulos, D.; Upmacis, R. K. Crystal Structure of Hexakis(Dimethyl Sulfoxide-κ*O*)Cobalt(II) Bis[Trichlorido(Quinoline-κ*N*)Cobaltate(II)]. *Acta Crystallogr. Sect. E Crystallogr. Commun.* **2018**, *E74* (3), 309–312.

(9) Abu Ali, H.; Abu Shamma, A.; Kamel, S. New Mixed Ligand Cobalt(II/III) Complexes Based on the Drug Sodium Valproate and Bioactive Nitrogen-Donor Ligands. Synthesis, Structure and Biological Properties. *J. Mol. Struct.* **2017**, *1142*, 40–47.

(10) Cui, Y.; Zhang, X.; Zheng, F.; Ren, J.; Chen, G.; Qian, Y.; Huang, J. Two Mixed-Metal Carboxylate–Base Adducts. *Acta Crystallogr. C* **2000**, *C56* (10), 1198–1200.

(11) Cui, Y.; Zheng, F.; Huang, J. Tetrakis(μ-Phenylacetato-*O*:*O*')Bis[(Quinoline-*N*)Cobalt(II)]. *Acta Crystallogr. C* **1999**, *C55* (7), 1067–1069.

(12) Anacona, J. R.; Azocar, M.; Nusetti, O.; Rodriguez-Barbarin, C. Crystal Structure of the First SH-Containing Tetrahedral Cobalt(II) Complex, [Co(Quinoline)2(SH)2]. Superoxide Dismutase Activity. *Transit. Met. Chem.* **2003**, *28* (1), 24–28.

(13) Cui, Y.; Long, D.; Huang, X.; Zheng, F.; Chen, W.; Huang, J. *Chin J Struct Chem* **1999**, *18*, 9.

(14) Pan, T.-T.; Xu, D.-J. Quinolinium Trichloro(Quinoline-κ*N*)Cobaltate(II). *Acta Crystallogr. Sect. E Struct. Rep. Online* **2003**, *E60* (1), m56–m58.

(15) Oka, Y.; Inoue, K. Structures and Magnetic Properties of a New Cobalt(II) Linear Trimer with Phenylcinnamic Acid. *Chem. Lett.* **2004**, *33* (4), 402–403.

(16) Saber, M. R.; Dunbar, K. R. Ligands Effects on the Magnetic Anisotropy of Tetrahedral Cobalt

Complexes. *Chem Commun* **2014**, *50* (82), 12266–12269.

(17) Mirčeva, A.; Golič, L. Structure of Cobalt Diquinoline Diisothiocyanate. *Acta Crystallogr. C* **1990**, *C46* (6), 1001–1003.

(18) Chen, D.-Y.; Gao ,Hui; Hu ,Xue-Fu; Guo ,Xiang-Yang; Yang ,Fan; and Bai, Y. Synthesis, Crystal Structure, and Luminescent Properties of Two Ternary Complexes with Mixed Thiocyanate and Quinoline Ligands. *Synth. React. Inorg. Met.-Org. Nano-Met. Chem.* **2010**, *40* (2), 112–115.

(19) Ali, S.; Muryn, C. A.; Tuna, F.; Winpenny, R. E. P. Synthesis and Structural and Magnetic Characterisation of Cobalt(II) Complexes of Mixed Phosphonate-Antimonate Ligands. *Dalton Trans.* **2010**, *39* (40), 9588.

(20) Cui, Y.; Zheng, F.-K. Syntheses and Crystal Structures of Di-, Tri-and Tetra-Nuclear Cobalt (II)-Lanthanide (III) Carboxylate Complexes. *Chin J Struct Chem* **2001**, *20* (2), 112–119.

(21) Abrahams, B. F.; Elliott, R. W.; Hudson, T. A.; Robson, R. A New Class of Easily Generated $TCNQ^{2-}$-Based Coordination Polymers. *Cryst. Growth Amp Des.* **2010**, *10* (7), 2860–2862.

(22) Peppel, T.; Köckerling, M. Investigations on a Series of Ionic Liquids Containing the $[Co^{II}Br_3Quin]^-$Anion (Quin = Quinoline). *Cryst. Growth Amp Des.* **2011**, *11* (12), 5461–5468.

(23) Villa, A. C.; Guastini, C.; Porta, P.; Tomlinson, A. A. G. Complexes with Sulphur and Selenium Donor Ligands. Part 7. The Crystal and Molecular Structure of Bis(Diphenylphosphinodithioato)-Cobalt(II)–Quinoline (1/1). *J Chem Soc Dalton Trans* **1978**, No. 8, 956–959.

(24) Meher, N. K.; Verma, P. K.; Geetharani, K. Cobalt-Catalyzed Regioselective 1,2-Hydroboration of N-Heteroarenes. *Org. Lett.* **2023**, *25* (1), 87–92.

(25) Cui, Y.; Chen, J.-T.; Long, D.-L.; Zheng, F.-K.; Cheng, W.-D.; Huang, J.-S. Preparation, Structure and Preliminary Magnetic Studies of Tri- and Tetra-Nuclear Cobalt–Lanthanide Carboxylate Complexes. *J. Chem. Soc. Dalton Trans.* **1998**, No. 18, 2955–2956.

(26) Korchagin, D. V.; Shilov, G. V.; Aldoshin, S. M.; Morgunov, R. B.; Talantsev, A. D.; Yureva, E. A. Halogen Atom Effect on the Magnetic Anisotropy of Pseudotetrahedral Co(II) Complexes with a Quinoline Ligand. *Polyhedron* **2015**, *102*, 147–151.

(27) Lomjanský, D.; Varga, F.; Rajnák, C.; Moncoľ, J.; Boča, R.; Titiš, J. Redetermination of Zero-Field Splitting in [Co(Qu)2Br2] and [Ni(PPh3)2Cl2] Complexes. *Nova Biotechnol. Chim.* **2016**, *15* (2), 200–211.

(28) Golič, L.; Mirčeva, A. Structure of Dichlorodiquinolinecobalt(II): Isomorphism with the Analogous Co, Ni and Zn Halogenides. *Acta Crystallogr. C* **1988**, *C44* (5), 820–822.

(29) Wang, W.; Huang, D.; Zhu, H.; Chen, C.; Liu, Q. Monoclinic Form of Dichlorobis(Quinoline-*N*)Cobalt(II). *Acta Crystallogr. Sect. E Struct. Rep. Online* **2001**, *E57* (12), m587–m588.

(30) Li, L.; Chen, S.; Zhou, R.-M.; Bai, Y.; Dang, D.-B. A Novel Cobalt (I) Coordination Polymer with Mixed Thiocyanate and Quinoline Ligands: Crystal Structure, Magnetism and Luminescent

Properties. *Spectrochim. Acta. A. Mol. Biomol. Spectrosc.* **2014**, *120*, 401–404.

(31) Cui, Y.; Chen, G.; Ren, J.; Qian, Y.; Huang, J. Syntheses, Structures and Magnetic Behaviors of Di- and Trinuclear Pivalate Complexes Containing Both Cobalt(II) and Lanthanide(III) Ions. *Inorg. Chem.* **2000**, *39* (18), 4165–4168.

(32) Lutsenko, I. A.; Kiskin, M. A.; Nikolaevskii, S. A.; Starikova, A. A.; Efimov, N. N.; Khoroshilov, A. V.; Bogomyakov, A. S.; Ananyev, I. V.; Voronina, J. K.; Goloveshkin, A. S.; Sidorov, A. A.; Eremenko, I. L. Ferromagnetically Coupled Molecular Complexes with a $Co^{II}_2Gd^{III}$ Pivalate Core: Synthesis, Structure, Magnetic Properties and Thermal Stability. *ChemistrySelect* **2019**, *4* (48), 14261–14270.

(33) Wang, R.; Kim, D.; Park, S. Selective Cascading Hydroboration of *N*-Heteroarenes via Cobalt Catalysis. *ACS Catal.* **2024**, *14* (5), 3582–3595.
